# High Functional Diversity in Mycobacterium tuberculosis Driven by Genetic Drift and Human Demography

**DOI:** 10.1371/journal.pbio.0060311

**Published:** 2008-12-16

**Authors:** Ruth Hershberg, Mikhail Lipatov, Peter M Small, Hadar Sheffer, Stefan Niemann, Susanne Homolka, Jared C Roach, Kristin Kremer, Dmitri A Petrov, Marcus W Feldman, Sebastien Gagneux

**Affiliations:** 1 Department of Biology, Stanford University, Stanford, California, United States of America; 2 Institute for Systems Biology, Seattle, Washington, United States of America; 3 Bill and Melinda Gates Foundation, Seattle, Washington, United States of America; 4 Forschungszentrum Borstel, National Reference Center for Mycobacteria, Borstel, Germany; 5 Seattle Children's Hospital Research Institute, Seattle, Washington, United States of America; 6 Mycobacteria Reference Unit (CIb-LIS), National Institute of Public Health and the Environment, Bilthoven, The Netherlands; 7 MRC National Institute for Medical Research, London, United Kingdom; New York University School of Medicine, United States of America

## Abstract

Mycobacterium tuberculosis infects one third of the human world population and kills someone every 15 seconds. For more than a century, scientists and clinicians have been distinguishing between the human- and animal-adapted members of the M. tuberculosis complex (MTBC). However, all human-adapted strains of MTBC have traditionally been considered to be essentially identical. We surveyed sequence diversity within a global collection of strains belonging to MTBC using seven megabase pairs of DNA sequence data. We show that the members of MTBC affecting humans are more genetically diverse than generally assumed, and that this diversity can be linked to human demographic and migratory events. We further demonstrate that these organisms are under extremely reduced purifying selection and that, as a result of increased genetic drift, much of this genetic diversity is likely to have functional consequences. Our findings suggest that the current increases in human population, urbanization, and global travel, combined with the population genetic characteristics of M. tuberculosis described here, could contribute to the emergence and spread of drug-resistant tuberculosis.

## Author Summary

Tuberculosis remains a worldwide public health emergency. The emergence of drug-resistant forms of tuberculosis in many parts of the world is threatening to make this important human disease incurable. Even though many resources are being invested into the development of new tuberculosis control tools, we still do not know the extent of genetic diversity in tuberculosis bacteria, nor do we understand the evolutionary forces that shape this diversity. To address these questions, we studied a large collection of human tuberculosis strains using DNA sequencing. We found that strains originating in different parts of the world are more genetically diverse than previously recognized. Our results also suggest that much of this diversity has functional consequences and could affect the efficacy of new tuberculosis diagnostics, drugs, and vaccines. Furthermore, we found that the global diversity in tuberculosis strains can be linked to the ancient human migrations out of Africa, as well as to more recent movements that followed the increases of human populations in Europe, India, and China during the past few hundred years. Taken together, our findings suggest that the evolutionary characteristics of tuberculosis bacteria could synergize with the effects of increasing globalization and human travel to enhance the global spread of drug-resistant tuberculosis.

## Abstract 3

DNA sequence analysis of a global collection of*M. tuberculosis* strains reveals high functional diversity, severely reduced selective constraint, and global spread through both ancient and recent human migrations.

## Introduction

Mycobacterium tuberculosis is a gram-positive bacterium and the causative agent of human tuberculosis. The worldwide emergence of multidrug-resistant strains of M. tuberculosis is threatening to make tuberculosis incurable [1]. Although renewed efforts are being directed towards the development of new tools to better control tuberculosis [2], much about the evolution of this obligate human pathogen remains unknown [3].

In 1898, Harvard pathologist Theobald Smith demonstrated that tubercle bacilli isolated from humans differed significantly from bacilli isolated from cattle in their capacity to cause disease in different animal species [4]. Eventually, the two bacilli were granted separate species status, with M. tuberculosis designating the typical human pathogen, and Mycobacterium bovis referring to the bovine form [5]. Because M. bovis has the capacity to cause disease in a variety of animal species, including humans, it was originally thought to exhibit a much broader host range than M. tuberculosis. However, recent comparative genomic analyses have revealed such a high degree of genetic diversity in M. bovis that modern population geneticists now consider the species to be comprised of several ecotypes, each of which is adapted to particular animal host species [6–10]. Some of these ecotypes have been given distinct species designations. For example, Mycobacterium microti is a pathogen of voles [11], Mycobacterium pinnipedii a pathogen of seals and sea lions [12], and Mycobacterium caprae a pathogen of goats [13].

By contrast, the human-adapted members of the M. tuberculosis complex (MTBC) have traditionally been assumed to be essentially identical. This notion was primarily driven by the results of early studies that revealed very low levels of DNA sequence variation in human MTBC [14,15]. More recent surveys of global strain collections show that in fact human MTBC consists of separate strain lineages associated with different regions of the world [16–20]. However, all of these studies have important limitations such that the actual phylogenetic distances and relative genetic diversities within and between mycobacterial lineages have not been determined [21,22]. Specifically, the study by Brudey et al. [17] used the standard molecular epidemiological method known as spoligotyping to determine the global population structure of M. tuberculosis. However, because this technique indexes genetic diversity based on the presence or absence of a repetitive sequence at a single locus (the “direct repeat region” of M. tuberculosis), which is prone to convergent evolution, this technique is of limited use for phylogenetic and population genetic analyses [16,18,20,21,23]. The study by Baker et al. [16] used a multilocus sequencing approach to study M. tuberculosis diversity, but because just seven genes were analyzed, only a small number of phylogenetically informative single nucleotide polymorphisms (SNPs) were identified. In the studies by Gutacker et al. [20] and Filliol et al. [18], the authors used a very similar approach: they compared the full genome sequences of MTBC strains available at the time and identified a series of synonymous SNPs, which they used to genotype large collections of strains. However, such approaches are known to lead to so-called phylogenetic discovery bias and distorted phylogenetic inference [22,24,25]. In our previous study [19], we used genomic deletions (large sequence polymorphisms) to analyze a global collection of strains. Even though we were able to use these deletions to classify strains unambiguously, genetic distances based on genomic deletions are difficult to interpret [3,21]. Finally, because of the inherent limitations of the molecular markers used in all of the studies reviewed above, the evolutionary processes that shape strain diversity in MTBC have not been adequately investigated; generally, actual DNA sequence data are preferred for phylogenetic and population genetic analyses [26,27].

Here we report our in-depth analyses of a large set of coding sequence data from a global collection of MTBC strains. These analyses reveal that the human-adapted members of MTBC are more genetically diverse than generally recognized. We also demonstrate that genetic drift is likely to be an important evolutionary force generating diversity in MTBC, and that this diversity can be linked to changes in human demography and to both ancient and recent human migrations.

## Results and Discussion

### The Global Phylogeny of M. tuberculosis

We investigated the genetic diversity within MTBC using seven megabases of DNA sequence data that we generated from a representative collection of 108 MTBC strains (Table S1). This collection included 99 human-adapted strains that were selected to represent the broadest geographic and genetic diversity from a global collection of 875 strains characterized previously by the analysis of deletions across the genome [19]. An additional seven strains were selected to represent four animal-adapted ecotypes, including M. bovis, M. microti, M. pinnipedii, and *M. caprae*. We also included the vaccine strain M. bovis BCG Pasteur and one strain of Mycobacterium canettii as our predicted outgroup. M. canettii is formally considered part of MTBC. However, in this study we use “MTBC” to refer to all other members of MTBC, excluding M. canettii. M. canettii strains have been shown to be more distantly related to the remaining MTBC than any two other MTBC strains are to each other [28]. For each of the 108 strains included in the study, we determined the DNA sequence of 89 genes, which together corresponded to 65,829 base pairs per strain, or 1.5% of the ∼4.4 Mbp genome of MTBC (Tables S2 and S3, Figure S1). These 89 genes comprised housekeeping genes and antigens analyzed in the early sequencing studies mentioned above [14,15], as well as genes of special interest, including putative new drug targets [29–31], genes believed to be involved in latency and reactivation [32,33], DNA repair genes [34], genes encoding a novel bacterial secretion system [35,36], and novel antigens [37,38]. Although the selection of these 89 genes was not random, the genes are distributed fairly uniformly around the MTBC chromosome, thus covering all the main parts of the MTBC genome (Figure S1).

**Figure 1:**
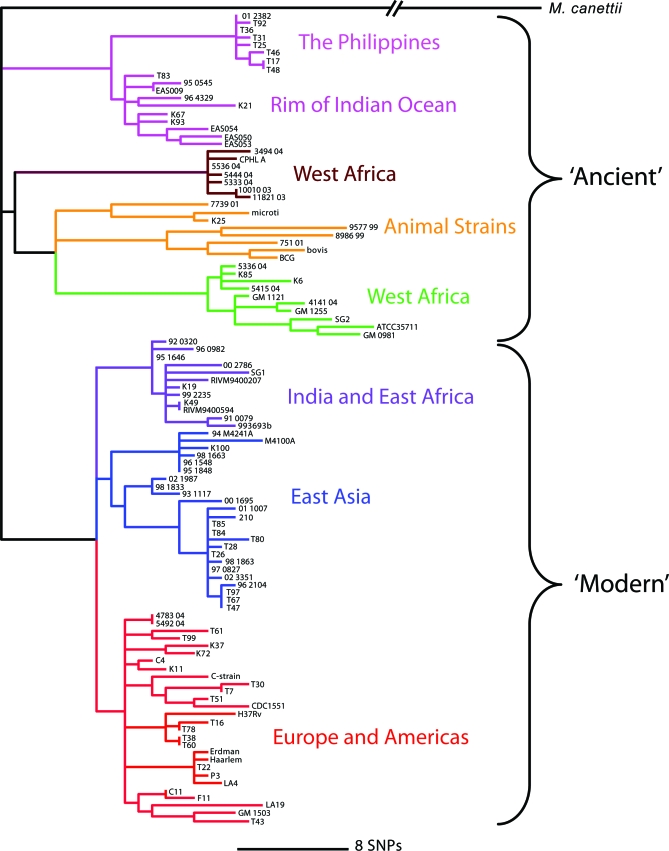
Maximum Parsimony Phylogeny of M. tuberculosis Complex Using 89 Concatenated Gene Sequences in 108 Strains The branches are colored according to the main lineages defined previously based on our genomic deletion analysis, except for the animal-adapted strains, which are indicated in orange. The main clades are labeled according to their dominance in particular geographic areas. The branch leading to M. canettii has been truncated in the figure because of the large numbers of changes that separate this hypothesized outgroup from the rest of the phylogeny (Table S4). Ancient and modern strain lineages are indicated. The green and brown lineages correspond to strains traditionally known as M. africanum [21].

We first used the concatenated DNA sequences of the 89 genes for each of the 108 strains and conducted a maximum parsimony analysis. Our analysis produced a single phylogenetic tree with a homoplasy index of 0.0043 (Figure 1). This phylogenetic tree is completely congruent with the one we constructed using the neighbor-joining method (Figure S2) as well as with the deletion-based analysis we reported previously (Figure 1, Table S1) [19]. The negligible degree of homoplasy observed in our sequence-based tree, and the fact that our sequence-based and deletion-based trees are congruent further supports the highly clonal population structure of MTBC [39,40]. The primary branches of our sequence-based tree are also consistent with earlier studies that classified M. tuberculosis into “ancient” and “modern” forms based on the presence or absence of a genomic deletion known as TbD1 [8]. However, because our new sequence data allow us to better interpret genetic distances between lineages, we find that the difference between ancient and modern MTBC is more pronounced than one would assume on the basis of the presence of a single genomic deletion (Figure 1).

### Human-Adapted MTBC Is More Genetically Diverse than Assumed

**Figure 2:**
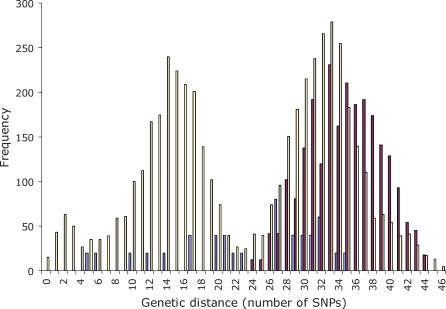
Frequency of Pairwise Genetic Distances Among 108 Strains of the MTBC Pairwise comparisons of genetic distances between human-adapted strains are indicated in yellow, those between animal-adapted strains in blue, and those between human- and animal-adapted strains in red. For better illustration, the frequencies of genetic distances in the animal-to-animal strain comparisons and the animal-to-human strain comparisons were multiplied by 20 and 3, respectively. However, for the statistical analysis (see main text), the actual frequencies were used.

We further compared the genetic distances between different strain lineages using our new sequence data. Overall, our analysis reveals greater genetic diversity of the human-adapted organisms than previously appreciated. Specifically, our sequence-based phylogeny shows that all the animal-adapted members of MTBC form an ingroup relative to the rest of the phylogeny. This suggests that even though these animal strains belong to four distinct ecotypes adapted to distinct animal host species, they represent only a proportion of the genetic diversity found in all of the human-adapted MTBC (Figure 1). In fact, the average pairwise genetic distance among animal-adapted strains is equal to the average distance among human-adapted strains (23.61 versus 23.65 differences, respectively; Figure 2). Additionally, the distribution of all possible pairwise genetic distances within the human-adapted group was not significantly different from that within the animal-adapted group (Mann–Whitney rank sum test, *p* = 0.74). Taken together, this evidence shows that the genetic diversity among the human-adapted members of MTBC is just as pronounced as that among the animal-adapted strains tested. Although the human MTBC strains used in this study are not a true population survey because they were selected to maximize either diversity or geographical distribution, these observations suggest that the genetic diversity among the human-adapted members of MTBC is just as pronounced as that among the animal-adapted strains. This is especially surprising given the fact that the latter belong to four distinct ecotypes adapted to distinct animal host species.

### Purifying Selection Is Severely Reduced in MTBC

Next, we used our sequence dataset to estimate the role of purifying selection in the evolution and genetic diversity of MTBC. One commonly used method of examining the degree of purifying selection acting on sequences is to calculate the ratio of the rates of nonsynonymous and synonymous changes (dN/dS). In the absence of selection this ratio is expected to near unity. Purifying selection is expected to reduce this ratio, while positive selection is expected to increase it.

We discovered a total of 488 SNPs in our 108 strains. The M. canettii strain differed from each of the other MTBC strains at 129–145 sites (0.2% of the examined sites, Table S4), while the maximum number of SNPs between any two other MTBC strains was 46 (0.07% of the examined sites). This supports the use of M. canettii as a closely related outgroup. The comparison of the proteins in the M. canettii strain with the majority-rule consensus of the proteins in the remaining MTBC strains revealed 125 nucleotide differences, of which 43 were nonsynonymous. This corresponds to the ratio of the rates of nonsynonymous and synonymous changes (dN/dS) of 0.18. This ratio is similar to that previously observed between M. tuberculosis and the relatively more distantly related Mycobacterium avium (0.17) [41]. It is also similar to ratios we obtained by pairwise comparisons of the two fully sequenced M. avium strains: M. avium 104 and *M. avium paratuberculosis* (dN/dS = 0.17), and for the two fully sequenced strains: *Mycobacterium sp.* JLS and *Mycobacterium sp.* MCS (dN/dS = 0.15). This appears to represent the general dN/dS within the Actinobacteria, as we conclude from an examination of pairwise genome-wide comparisons of different fully sequenced Actinobacteria genomes (unpublished data). It thus appears that the strength of selection acting on M. canettii may be similar to that acting on other Mycobacteria and Actinobacteria.

Out of the 370 SNPs that segregated among the remaining 107 MTBC strains (i.e., excluding M. canettii; Table S5), 231 (62%) were nonsynonymous and 139 (38%) synonymous. The average pairwise dN/dS ratio for the MTBC strains was 0.57. This is substantially higher than that in most other bacteria [41] and is significantly higher than that observed for the SNPs specific to M. canettii (G-test, *p* < 0.0001).

The high dN/dS in MTBC could be a consequence of diversifying selection driven by host immunity. We addressed this possibility by comparing the ratio of nonsynonymous to synonymous SNPs in surface-exposed or excreted, virulence, and housekeeping genes (Table S2). Contrary to what we would expect if immune selection was responsible for the high dN/dS in MTBC, we found that all three gene classes had comparable ratios of nonsynonymous to synonymous SNPs (Table S6), similar to the situation reported for Salmonella typhi [42]. It is therefore unlikely that diversifying selection explains the high ratio of dN/dS unless housekeeping proteins, virulence proteins, and surface- exposed or secreted proteins are all equally and strongly exposed to immune surveillance and are all involved in the creation of antigenic variation. This latter possibility seems unlikely because, even though M. tuberculosis is an intracellular pathogen, during various stages of the natural history of human tuberculosis (for example transmission and infection processes, and cavitary and disseminated disease) bacilli are in fact located extracellularly. Furthermore, there is increasing evidence that M. tuberculosis interacts with both the humoral and the cellular immune system as evidenced by antibody-based serological recognition of M. tuberculosis antigens in patient sera [38]. This suggests that surface-exposed or excreted gene products in MTBC should, in principle, be under stronger immune surveillance than structural genes.

**Figure 3:**
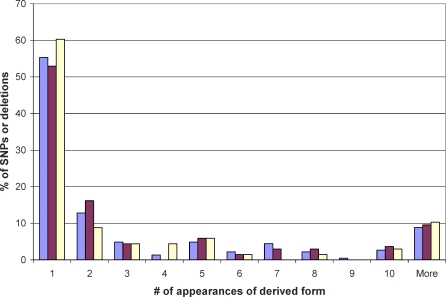
Frequency Distribution of Nonsynonymous Mutations, Synonymous Mutations, and Genomic Deletions in MTBC Strains For each site that underwent a mutation within MTBC we observed two states: the derived (or mutated) state, and the ancestral state. For synonymous and nonsynonymous mutations we deduced the ancestral state based on the sequence of the outgroup strain M. canettii. For the genomic deletions (originally described in [44]), the ancestral state is the non-deleted state and the deleted state is derived. The frequencies depicted here are those of the mutated states. All three types of mutations show similar frequency distributions, which suggests that most nonsynonymous mutations and deletions are not slightly deleterious but rather selectively neutral within MTBC. Blue, nonsynonymous mutations; red, synonymous mutations; yellow, genomic deletions.

In other organisms, high dN/dS ratios are often considered to indicate a reduction in purifying selection [26,27]. However, such high dN/dS values may also stem from the close relatedness of the MTBC strains. Rocha et al. pointed out that dN/dS is often higher in cases in which the organisms being compared are very closely related [43]. For such closely related organisms the dN/dS ratio may be elevated because slightly deleterious nonsynonymous mutations that are destined to be lost during long periods of time may not yet have been removed by purifying selection [43]. If dN/dS within MTBC is inflated because of nonsynonymous mutations being slightly deleterious, we expect the frequency distribution for nonsynonymous SNPs to be skewed towards low frequencies when compared with synonymous SNPs. Using our large dataset of sequences it was possible to test this directly. We found that although the frequency distribution of nonsynonymous SNPs was highly skewed towards low frequencies, this was equally true for the synonymous SNPs (Figure 3), with no difference in the proportion of singletons in the two types of SNPs (G-test, *p* = 0.66). Similarly, when we examined the frequency of 68 genomic deletions in 100 clinical MTBC strains reported earlier [44], we found that these deletions were also not more likely to occur as singletons when compared with the synonymous SNPs found here (G-test, *p* = 0.32). These results indicate that neither nonsynonymous SNPs nor deletions show any evidence of being slightly deleterious within MTBC (i.e., they behave as selectively neutral). Second, if slightly deleterious mutations contribute to the increased dN/dS in MTBC, we expect dN/dS to be lower among common SNPs, as slightly deleterious polymorphisms are less likely to reach high frequencies. However, we found that the proportion of nonsynonymous SNPs among the 75 SNPs that were present in more than five strains was no different from the proportion in the entire dataset (63% compared with 62%, G-test, *p* = 0.97). To further test our hypothesis, we compared the ratio of nonsynonymous to synonymous SNPs in different parts of our phylogenetic tree (Figure 1). We found no significant difference in this ratio between internal and external phylogenetic branches or between the ancient and modern MTBC lineages (Table S6). These results together indicate that, contrary to the suggestion of Rocha et al. [43], the high dN/dS values within MTBC are not an artifact of the close relatedness of the MTBC strains, but are in fact likely to be the result of a reduction in selective constraint.

To further probe the observed reduction in selective constraint, we classified different positions within the studied proteins according to their patterns of conservation in all ten fully sequenced mycobacterial species that are distantly related to the MTBC/M. canettii strains. We first searched for orthologs of the 89 sequenced genes in these ten mycobacterial species. For 62 of the genes we could find orthologs in at least five of the ten species (Table S2). We aligned the protein sequences of these 62 genes in the mycobacterial species in which they were present (excluding MTBC/M. canettii orthologs) and examined the level of conservation at each alignment position. The amino acid positions were then divided into two groups: (i) “conserved” positions—positions that either have identical amino acids in all the examined distant mycobacterial species or vary only among biochemically similar amino acids, and (ii) “variable” positions—the least constrained positions that show some radical amino acid changes (see Methods). 64% of positions in these proteins were conserved and 36% were variable (Table 1). We expect that mutations at the conserved positions should have stronger functional effects and be more deleterious on average than mutations at the variable positions. Consistent with this view, nonsynonymous changes specific to M. canettii predominantly fall into variable positions (72%, G-test, *p* < 0.0001, Table 1). We expect that weakened purifying selection in MTBC should allow more amino acid changes in MTBC to be observed at the conserved (i.e., constrained) positions. As expected, in contrast to the changes in M. canettii, the majority (58%) of amino acid mutations in MTBC fall into the conserved positions (Table 1). In fact, the proportions of amino acid mutations in MTBC that fall into variable and conserved positions is not significantly different from that expected if purifying selection in MTBC was no longer making a distinction among mutations in these two classes of sites (G-test, *p* = 0.1). A different way to look at this is to calculate a measure similar to dN/dS that compares the rates of variable and conserved amino acid changes. *P*_var_ can be defined as the number of changes that fall at variable sites divided by the total number of variable sites. Similarly *P*_cons_ is defined as the number of changes at conserved sites divided by the total number of conserved sites. *P*_cons_/*P*_var_ equals 0.22 for the *M. canettii* changes and 0.77 for the MTBC changes. This analysis further underscores the low level of purifying selection in MTBC.

**Table 1:**
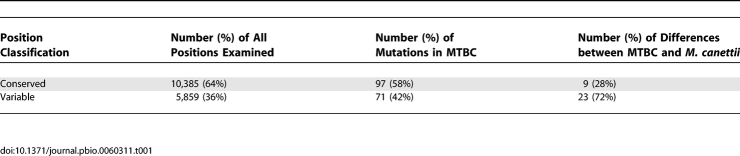
Classification of Mutations According to Degree of Conservation in Distantly Related Mycobacterial Species

### The Observed Reduction in Selection Is Not Specific to the 89-Gene Dataset Used

To evaluate whether the 89 genes we selected were indeed representative of the whole M. tuberculosis genome, we repeated some of our analyses using completed MTBC genomes. Currently, full genomic sequences are available for six MTBC strains: M. tuberculosis H37Rv, M. tuberculosis H37Ra, M. tuberculosis F11, M. tuberculosis CDC1551, M. bovis, and M. bovis BCG Pasteur 1173P2. We conducted genome-wide pairwise comparisons of the DNA sequences of all orthologous protein pairs between the H37Rv strain and the remaining five strains. This allowed us to obtain the number of synonymous and nonsynonymous differences for all orthologs across the genome. As strains belonging to MTBC show very low levels of diversity, it is impossible to use pairwise comparisons to calculate dN and dS for specific genes. However, it is possible to sum the mutations across the genome and examine whether the ratios of nonsynonymous and synonymous mutations that we found for the 89 genes in our dataset are representative of the entire genome. For the vast majority of orthologs pairs we found zero to two differences between the H37Rv strain and any of the other five strains (>97% for all comparisons). However, in each comparison there was a small number of genes (less than ten) that were clearly outliers. These genes showed a much higher number of differences than the genome in general. Many of these genes, such as *pks12* and members of the PE and PPE gene families, were previously implicated as involved in antigenic variation [45,46] and may be subject to diversifying selection. Alternatively, they could represent rare examples of recombination or horizontal gene transfer in MTBC, for which some evidence has been presented [47,48]. These genes do not seem to be representative of the genome as a whole, given the small number of mutations in all other genes and the large number of mutations within these genes; including them may skew the analysis. We therefore removed these genes prior to summing the mutations. For all five comparisons the ratio of nonsynonymous to synonymous differences was very similar to the ratio found within our 89 gene dataset (Table 2, G-test, *P* > 0.92 for all five comparisons). It thus appears that the reduced selection we observed is not limited to the genes within our dataset.

**Table 2:**
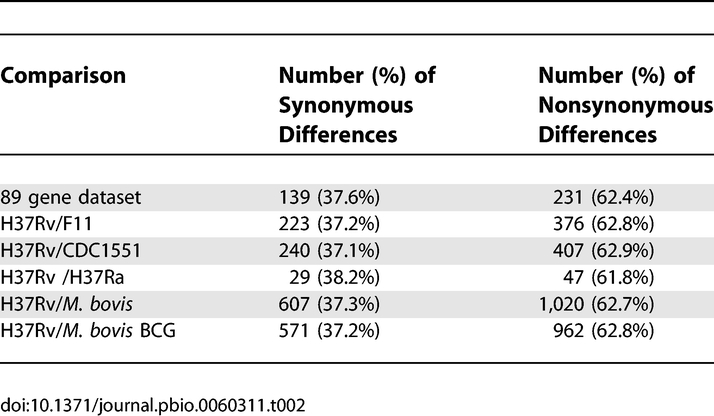
Synonymous and Nonsynonymous MTBC Differences in the 89 Gene Dataset and in Genome-Wide Pairwise Comparisons

This genome-wide analysis also provides a further indication that the observed high ratio of dN/dS is not the result of immune surveillance. Similarly to the majority of the genes in the genome, and in sharp contrast to *pks12* and some genes of the PE and PPE families that are known to be under immune surveillance, the 89 genes we sequenced show only zero to two differences between the H37Rv strain and any of the other five fully genome sequenced strains. This, in addition to our finding that the high dN/dS ratio is not specific to the 89 analyzed genes, but rather is characteristic of the genome as a whole, indicates that immune surveillance is not likely to explain the observed high dN/dS values, unless it affects the entire tuberculosis genome. Furthermore, the fact that some genes that are known to be under immune surveillance show much higher levels of diversity than most other genes in the genome further indicates that most genes are not affected to such a high extent by immune surveillance.

We further used the six fully sequenced MTBC genomes to calculate *P*_cons_/*P*_var_ at a genome-wide level. We created multiple sequence alignments of all of the annotated proteins of the H37Rv strain that have clear orthologs in the other five MTBC genomes. We also searched for orthologs of these sequences in the ten more distantly related fully sequenced mycobacterial strains mentioned earlier. For this analysis, we selected genes for which we could find clear orthologs in all of the six MTBC strains and create multiple sequence alignments in these six strains that contained no gaps. We also required that we find orthologs for these genes in at least five of the more distantly related mycobacterial strains. 1,970 genes filled these requirements. We could find no SNPs within the six MTBC strains for 1,289 of these genes and thus removed them from the analysis. We further removed four genes because they were clearly outliers and had well over ten SNPs each, whereas the vast majority of genes had three or fewer SNPs. These four genes include three-members of the PE and PPE protein families, which have been implicated as being involved in antigenic variation [45], as well as the *pks12* gene, which has been shown to produce a polyketide that is an important T cell antigen [46]. After removing these four genes, we were left with 677 genes in which there was at least one MTBC SNP. Within these genes 145,020 of the sites were conserved in the distantly related Mycobacteria (49%) and 149,728 were variable (51%). 448 of the MTBC SNPs found within these genes were at conserved sites (47%) and 515 of the SNPs fell at variable sites (53%). As was the case for the 89 genes in our dataset, there is no significant difference between the proportion of SNPs falling in variable and conserved sites and the random proportion we would expect if selection were not discriminating between the two types of sites (G-test, *p* = 0.1). Furthermore, when we calculated the genome-wide *P*_cons_/*P*_var_ we found it was even higher (0.9) than for the 89 gene analysis (0.77). Taken together, our genome-wide analyses show that the severe reduction in purifying selection we observed is not specific to the 89 genes in our main dataset. Rather there appears to be a genome-wide reduction in purifying selection in MTBC.

### Much of MTBC Diversity Has Functional Consequences

There are two possibilities regarding the nature of the observed reduced constraint. One possibility is that the reduction in purifying selection is pathway specific and that it affects only specific mutations that no longer have functional consequences as a result of changes in the ecological requirements of the MTBC strains. If this were true, one would expect to see a relaxation of purifying selection in only a subset of genes, and not in housekeeping genes that are generally universally required. However, the reduction of purifying selection within the MTBC appears to be genome-wide and occurs across different classes of genes, including housekeeping genes. This suggests the alternative possibility, in which a genome-wide reduction in the efficacy of purifying selection has occurred. The weakened selective constraint in MTBC appears to allow amino acid changes that would be removed by purifying selection in M. canettii and other mycobacteria to persist within MTBC. Such changes are likely to have deleterious functional consequences in M. canettii, as otherwise they would not have been removed by selection*.* Most of these mutations are still likely to have similar functional effects in the other MTBC strains. However, because of the reduced efficiency of selection they are more likely to persist.

It is possible to roughly estimate the proportion of the differences within MTBC that are likely to carry such functional consequences. On the basis of our 89 gene dataset, we found that in *M. canettii,* 23 mutations fell into variable sites and nine, or ∼2.6 times fewer, fell into conserved sites. Had selection been as strong in MTBC as it is in *M. canettii,* and assuming conservatively that the mutations at the variable sites are entirely neutral, we would expect 2.6 times fewer mutations to be observed at constrained sites within MTBC as well. Considering that there are 71 mutations at variable sites in MTBC we would expect ∼27 mutations at conserved sites within MTBC. Instead, we find 97 such mutations. This indicates that close to 72% of mutations at conserved sites (42% of the amino acid mutations overall) within MTBC would have been removed by selection in M. canettii. Because such amino acid mutations are likely to affect protein function, this suggests that about 40% of amino acid changes in MTBC have functional consequences.

To put this in context, we can consider the number of nonsynonymous differences found between the fully sequenced M. tuberculosis H37Rv and other two fully sequenced strains of M. tuberculosis present in our dataset: CDC1551 and F11. We considered only genes that are clear orthologs, could be aligned across their entire protein sequence, and had no gaps in their DNA alignment that could represent frameshift events. Again, we removed genes that were clear outliers with respect to the number of mutations they contain (more than ten mutations).Within the remaining protein pairs, we found 423 and 410 amino acid differences between H37Rv and CDC1551, and H37Rv and F11, respectively. Note that the genome-sequenced strains do not represent the full diversity within MTBC, because they all belong to the same strain lineage (red in Figure 1). However, if the sequenced proteins are representative of the genome as a whole, these numbers and the diversity in our dataset can be used to conservatively estimate the range of functional amino acid differences between an average pair of MTBC strains. For the 89 genes we sequenced, the number of differences between strain pairs ranged from zero to 46 (Figure 2), with an average of 25. For these genes we find 17 differences between H37Rv and CDC1551, and 13 differences between H37Rv and F11. From these numbers we can estimate conservatively that an average pair of MTBC strains should have around 300 functional differences between them. At the same time, the least diverged strains may have close to no functional differences whereas the most diverged strains may differ at around 500 functional sites (see Methods). Furthermore, because we used a highly conservative estimate of the number of amino acid differences per genome and took only point mutations into account, this is likely to be a highly conservative estimate of the number of functional differences.

**Figure 4:**
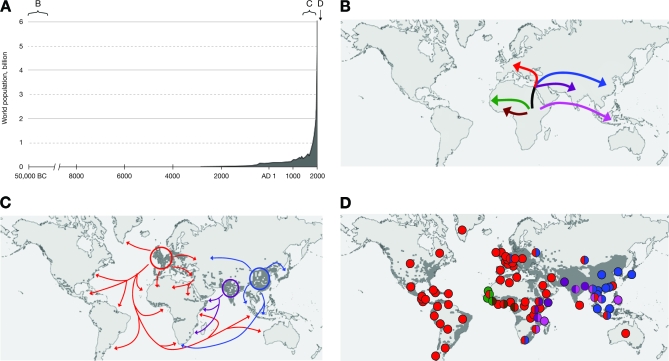
Out-Of-And-Back-To-Africa Scenario for the Evolutionary History of Human-Adapted MTBC (A) Global human population size during the last 50,000 y. The letters above the graph indicate the time periods corresponding to (B), (C), and (D), respectively (data source: http://en.wikipedia.org/wiki/Image:Population_curve.svg). (B) Hypothesized migration out of Africa of ancient lineages of MTBC. Colored arrows correspond to the six main human-adapted MTBC lineages shown in Figure 1. The hypothesized common ancestor of the three modern lineages (in red, purple, and blue) is indicated in black. (C) Recent increase of global human population. Each dark grey dot corresponds to 1 million people (data source: http://www.pbs.org/wgbh/nova/worldbalance/numb-nf.html). The population increase was strongest in Western Europe, India, and East Asia (Figure S3). These three geographic regions are each associated with one of the three modern MTBC lineages (red, purple, and blue). Recent human migration, trade, and conquest have promoted global spread of these modern MTBC lineages. (D) The human population has reached 6 billion. The distribution of the six main human-adapted MTBC lineages we observe today is shown (colors correspond to Figures 1 and S2; based on data from [19,21]).

Taken together, our results strongly suggest that the high value of dN/dS in MTBC results from a significant reduction in purifying selection. This reduced selective constraint most probably results from a number of factors, all of which tend to reduce an organism's effective population size. These factors are high clonality (i.e., virtual absence of horizontal gene exchange) and the serial transmission bottlenecks characteristic of this pathogen. These serial bottlenecks are particularly tight in M. tuberculosis, because a single cell is enough to establish a new infection. Furthermore, the population structure of human MTBC is highly subdivided, both geographically (Figures 1 and 4D) and within the lungs of infected individuals. It has been shown that different lesions in lungs of tuberculosis patients can harbor genetically distinct subclones of a particular infecting strain [49]. Finally, clonal organisms are also prone to selective sweeps [6]. All of these features lead to small effective population sizes, in which the effects of random genetic drift are increased compared with those of natural selection [6,26,27]. Random genetic drift allows mutations to reach high frequencies that in organisms with large effective population sizes would be eliminated by natural selection. Such mutations are likely to affect protein function deleteriously (i.e., they are functional), which is why they are eliminated in other organisms. By contrast, the large amount of predicted functional variation we observed at conserved sites in MTBC shows that natural selection is not as efficient at removing deleterious mutations in this organism. This high functional diversity in MTBC reported here further stresses the need to consider strain diversity in the development of new tuberculosis diagnostics, drugs, and vaccines [21].

### An “Out-Of-And-Back-To-Africa” Scenario for Human MTBC

In addition to natural selection and genetic drift, migration and demographic changes can affect the generation and distribution of genetic diversity in a particular organism. Thus, we decided to use our new sequence-based diversity dataset to further explore the complex interactions between mycobacterial evolution and human population growth and travel [50,51]. Overall, our data support a hypothesized “Out-of-and-back-to-Africa” scenario of the phylogeography of human tuberculosis (Figures 1 and 4D) [19,21]. Specifically, most evidence indicates that MTBC originated in Africa. For example, Africa is the only continent in which all six major human MTBC lineages occur, and the deeply rooted lineages are present exclusively in West Africa (brown and green in Figures 1 and 4). An African origin for human tuberculosis is also consistent with a previous report on M. canettii and other so-called “smooth tubercle bacilli” that share a remote common ancestor with the other MTBC and which are primarily associated with countries at the Horn of Africa [28,52]. During hunter–gatherer times, human populations remained small and geographically scattered (Figure 4A), which favored within-family transmission and the resulting stable host–pathogen associations [19,21,39]; it has been postulated that the characteristic latency period in human tuberculosis might be an adaptation of M. tuberculosis to low host densities [53]. Thus, we would expect the ancient human migrations out of Africa ∼50,000 y ago [54] to be reflected in the population structure of human-adapted MTBC, similar to the structure that has been observed for Helicobacter pylori [55]. In our dataset, this is seen in the distribution of the three deeply rooted ancient strains. For example, the contemporary distribution of the ancient pink lineage around the Indian Ocean (Figures 1 and 4D) corresponds to the earliest spread of modern humans out of Africa [54]. Importantly, early migration out of Africa would have occurred over land as ocean-going transport technologies were developed only at a much later time point in human history [51,54].

### Ancient MTBC Spread by Land, Modern MTBC by Sea

We hypothesize that further overland migration seeded Western Europe, Northern India, and East Asia with what would become the three modern M. tuberculosis lineages (red, purple, and blue lineages in Figures 1 and 4B). Concurrently, human populations started to increase dramatically and disproportionately in these regions providing an ecological niche for the clonal expansion of these three strain lineages (Figures 4A, 4C, and S3). During the past few centuries with large-scale human migration, trade and conquest out of these three areas [51], these clades further spread to other areas of the world (Figure 4C). Specifically, the presence of Euro-American (red) strains on the American continent can be explained in terms of the exodus from the over-populated European cities to America at the end of the 19th century—a “vast movement that dwarfed all earlier migrations,” according to the historian William McNeill [51]. Furthermore, the presence of strains from this cluster in Africa, Asia, and the Middle East is in agreement with the cases of European colonization of the post-Columbian era. In a similar vein, the presence of strains from the Indian-East-African (purple) lineage in East Africa can be explained in terms of the recent history of migration to this region from the Indian subcontinent [56]. Finally, the presence of the East Asian/Beijing (blue) lineage in South Africa is best explained by the relatively recent import of slaves from Southeast Asia by Dutch colonialists and later immigration of Chinese workforces to South African gold mines [57,58]. Although some human trade routes go back several millennia and thus could have contributed to the spread of MTBC, it appears that the large-scale migration events discussed above, which were partially driven by the more recent and dramatic increases in population densities in Europe, India, and East Asia (Figures 4A and S3), were key for the spread of the modern MTBC lineages. Importantly, in contrast to the ancient migrations that followed mostly land routes, modern waves of spread increasingly followed routes of ocean-going ships (Figure 4C) to arrive to their contemporary distribution (Figure 4D).

**Figure 5:**
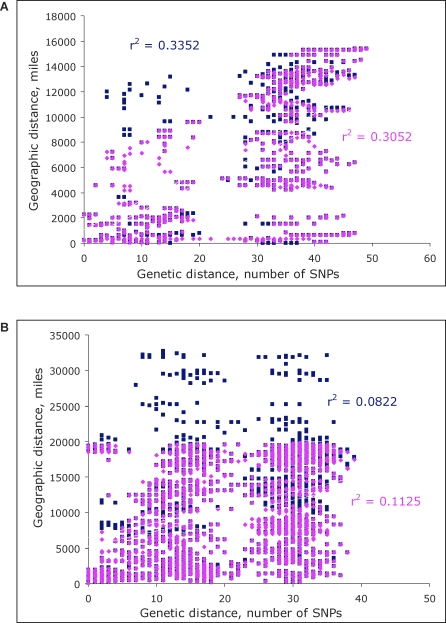
Correlations between Genetic and Geographic Distances in Ancient and Modern MTBC Strains To determine the route by which human-adapted MTBC strains were globally dispersed, we sought correlations between genetic and geographic distances in ancient (A) and modern (B) MTBC strains. The shortest distances between geographic locations via water routes (pink diamonds) or continental waypoints (i.e.*,* land routes; blue squares) are plotted against the corresponding genetic distances expressed as the number of SNPs. In ancient strains, the correlation using either route was highly statistically significant (*p* < 0.0001 for both), but the correlation using land routes was slightly stronger. By contrast in modern strains the correlation using water routes was stronger and more statistically significant compared with land routes (*p* < 0.0001 and *p* = 0.05, respectively). Significant *p* values and *r^2^* between 0 and 1 for all four analyses support the association between MTBC dispersal and human migration, which is consistent with early spread of MTBC out of Africa. The relative magnitude of *r* ^2^ for land routes versus water routes suggests that spread of ancient strains occurred over land (i.e.*,* through ancient human migration) and modern strains over water (i.e.*,* through recent historical migration, trade, and conquest).

The quantitative nature of our new diversity data permits a more rigorous analysis of this out-of-and-back-to-Africa hypothesis. Theory predicts that for an organism populating the world from a given point of origin, we will find a correlation between genetic variability and geographic distance traveled [59,60]. Thus, if our hypothesis is correct, we expect the genetic difference between any two strains to increase with geographic distance between where they originated. For all isolates, we used the haversine method with and without waypoints [59] to define pairwise distances over land routes and over water routes, respectively. Incorporation of waypoints into the calculation of geographic distances allows for the additional distances traveled via land routes during ancient human migrations. More direct geographic distances calculated without waypoints correspond to the water routes along which more recent human travel occurred once ocean-going ships became available. For each type of geographic distance, we computed the coefficient of linear correlation between geographic and genetic distances among ancient and modern strains, and tested its statistical significance. For all human-adapted MTBC strains we found a significant correlation between pairwise geographic and genetic distances, indicating that each group of strains carries a signal of the overall patterns of human demography and migration (Figure 5A and 5B). Furthermore, given the difference that we hypothesize between the migratory routes of ancient and modern strains we would expect that the correlation would be stronger for ancient strains spreading via land routes and for modern strains spreading via sea routes; this notion was supported by our statistical analysis (Figure 5A and 5B).

In summary, our quantitative analysis enabled by the sequence-based diversity data of MTBC strains supports the hypothesized relationship between the evolution of a very successful human pathogen and past human migrations. It is likely that more recent air travel might also be contributing to the global spread of MTBC variants, and part of the signal we detected for the spread of MTBC by water routes might in fact reflect more recent spread by air routes. However, as discussed above, it appears that changes in the global population structure of MTBC are linked to significant demographic changes and large-scale movements in human populations. On the long-term, increasing globalization and air travel could lead to homogenization of the global population structure of MTBC. Alternatively, it is conceivable that because of the distinct evolutionary trajectories of ancient and modern MTBC lineages, fundamental differences in the optimal virulence might limit the success of ancient lineages compared with modern strains [61]. In support of this possibility, a recent study in The Gambia reported that modern strains were three times more likely to cause rapid progression to active disease in secondary tuberculosis patients than strains that belonged to the ancient green lineage [62].

### Concluding Remarks

In conclusion, our analysis shows that human-adapted MTBC is more genetically diverse than traditionally assumed and is under greatly reduced selective constraint. This reduced selection pressure may have implications for the emerging epidemic of multidrug-resistant tuberculosis [1]. Drug-resistant strains of M. tuberculosis can exhibit fitness defects as a function of the specific drug-resistance-conferring mutation and strain genetic background [63,64]. However, reduced selection may allow strains harboring “costly” drug-resistance-conferring mutations to persist, even in the absence of antibiotics. Furthermore, persistence in the population of mutants resistant to a single drug, even if relatively unfit, may contribute to the emergence of multidrug-resistant organisms through the sequential acquisition of additional drug-resistance determinants. Given the complex linkage of this pathogen with past human demographic and migratory events, reduced selective constraint in M. tuberculosis could collude with the current increases in human population, urbanization, and global travel to exacerbate the worldwide epidemic of drug-resistant tuberculosis.

## Materials and Methods

### Bacterial strains.

A total of 108 mycobacterial strains were used in this study. A list with the detailed information on each strain is provided in Table S1. Ninety-nine strains were selected from our global collection of 875 strains from 80 countries previously analyzed by genomic deletion typing [19]. In addition, we included seven strains belonging to the four animal-adapted species of MTBC. These included two strains of M. bovis, two strains of M. caprae, two strains of M. microti, one strain of M. pinnipedii, and the vaccine strain BCG Pasteur. Finally, one strain of M. canettii was included as the hypothesized outgroup. Although we did not sample all the potential diversity among the animal-adapted mycobacteria, we included several representatives of four recognized species that exhibit different host preferences (i.e., four different ecotypes) [6–10].

### DNA sequencing.

Table S2 lists the information on the 89 genes analyzed in this study, and Figure S1 shows their position relative to the M. tuberculosis H37Rv genome. Eight of the 108 strains included in this study had complete or partial genome sequence data available in the public domain. These included the laboratory strain H37Rv (our reference strain) [65], *M. tuberculosis* strain CDC1551 [66], *M. bovis* strain AF2122/97 [5], M. bovis BCG Pasteur [67]*, M. tuberculosis* strain 210 (http://www.jcvi.org/), M. microti strain OV254 (http://www.sanger.ac.uk), and M. tuberculosis strains F11, C, and Haarlem (http://www.broad.mit.edu/seq/msc/). We used BLAST to identify sequence polymorphisms in these genomes relative to strain H37Rv.

For the remaining 100 strains, the 89 genes were PCR amplified and directly sequenced using the primers listed in table S3. PCR and sequencing primers were designed using PrimerSelect (Lasergene Inc.). DNA was amplified in a 96-well format and 25 μl reactions using a GeneAmp system 9700 thermocycler (Applied Biosystems). Unincorporated nucleotides and primers were removed by filtration with Multiscreen-PCR plates (Millipore). Sequencing reactions were performed with the BigDye Terminator (Applied Biosystems) and purified in Multiscreen plates (Millipore) with Sephadex (Amersham Biosciences). Sequence data were generated with an ABI 3730 XL automated sequencer (Applied Biosystems). For each gene, sequences were analyzed visually using the programs PHRED, PHRAP, and CONSED (University of Washington) and strain H37Rv as a reference. Only SNPs were considered in this analysis, and indels were ignored. SNPs that occurred in at least two strains were considered real. To exclude potential sequencing errors, SNPs that occurred only in a single strain were confirmed by sequencing of an independent PCR product, except for SNPs that occurred only in the M. canettii strain or in one of the strains with available whole genome sequences. Of the 20,500 initial sequencing reactions (205 primers in 100 strains; Table S3), 95 (<0.5%) failed to give interpretable results after several PCR and/or sequencing attempts. The corresponding gene sequences were assumed to be the same as the consensus in the final analysis.

### Phylogenetic analysis.

For phylogenetic inference, a data matrix was constructed, which included the concatenated sequences of the 89 genes for each of the 108 strains included in the study. We used DnaSP [68] to parse the raw data. Additional analyses were performed using a combination of the software PAUP [69], a mySQL database (http://www.mysql.com), several custom PERL scripts (http://www.perl.org), and a programmer's text editor TextMate (http://macromates.com). We first entered the NEXUS-formatted sequence output of DnaSP into PAUP, and ran a heuristic search for a maximum-parsimony tree with M. canettii strain K116 set as the outgroup. We then used PAUP's *describetrees* command to output the tree's branch lengths, its homoplasy index, character change lists, and the patristic distance matrix. The character change lists specify the tree branch where each single-nucleotide change occurred, according to the maximum parsimony criterion. The patristic distance matrix gives the number of differences separating any given pair of strains from each other. A neighbor-joining tree was constructed using the software MEGA 4 [70].

### Genetic distances between lineages.

We used a series of PERL scripts to parse, organize, and enter information about the maximum-parsimony tree into a mySQL database. To compute the distribution of pairwise genetic distances on the tree for animal–animal, human–human, and animal–human strain pairings, we extracted the lists of patristic distances for each kind of pairing from the mySQL database. We entered each list into a Microsoft Excel worksheet and constructed a series of overlapping histograms. To determine the significance of differences among the three distributions, we entered the patristic distance lists into the software package R (http://www.r-project.org/), and ran the Mann–Whitney rank sum test on each pair of distributions.

### dN/dS values.

The sequences of the 89 genes from each strain were concatenated and the resulting sequences were aligned using ClustalW. dN/dS within MTBC was calculated for the multiple alignment of the sequences of all the MTBC strains, using the program package DnaSP. dN/dS between MTBC and the outgroup strain M. canettii was calculated by estimating dN/dS between the majority-rule consensus sequence of MTBC and M. canettii. We compared M. canettii with the consensus sequence, rather than comparing M. canettii with each of the MTBC strains and averaging the result to eliminate the effect of evolution within MTBC on the comparisons. Pairwise dN/dS values were calculated using DnaSP according to the method of Nei and Gojobori [71]. In order to estimate whether the differences in dN/dS within MTBC and between MTBC and M. canettii are significant, we considered the fact that in all comparisons we are looking at the same exact sites. This allowed us to compare the number of nonsynonymous and synonymous mutations within MTBC with the same numbers between MTBC and M. canettii using a simple G-test of significance.

### Frequency of MTBC SNPs and deletions.

For synonymous and nonsynonymous mutations we deduced the ancestral state using the sequence of the outgroup strain M. canettii. Following this assignment we counted, for each SNP, the number of strains in which the derived state was observed. Deletion data were taken from the paper by Tsolaki *et al*. [44]*.*We assume that each deletion is the derived state, following Tsolaki *et al.* [44].

### Defining conserved and variable amino acid positions.

The full annotated proteomes of the following ten mycobacterial species were downloaded from the NCBI FTP server (ftp://ftp.ncbi.nlm.nih.gov/): M. avium 104*, M. avium paratuberculosis, M. gilvum* PYR-GCK*, M. sp.* JLS*, M. sp.* KMS*, M. sp.* MCS*, M. leprae, M. smegmatis* MC2 155*, M. ulcerans* Agy99*,* and *M. vanbaaleniis* PYR-1. To locate the orthologs of the 89 proteins included in our study and later for all the proteins of M. tuberculosis H37Rv in these ten bacterial genomes, we conducted a bidirectional FASTA [72] search of the protein sequences from the H37Rv strain of M. tuberculosis in each of the ten genomes. A M. tuberculosis protein was considered to have an ortholog in another Mycobacterium if it had a reciprocal best hit that could be aligned across at least 80% of its sequence. If a protein could be found in at least five of the ten mycobacterial genomes, we aligned the sequences of the proteins from each strain using ClustalW. Positions were divided into two groups: (i) conserved positions—positions that are either identical or that show only conservative amino acid differences and (ii) variable positions—positions that show some radical amino acid changes. Differences in amino acids were classified as conservative or radical based on the method of Zhang *et al.* [73]. According to this method amino acids are divided based on polarity and volume into six groups: special: C; neutral and small: A, G, P, S, and T; polar and relatively small: N, D, Q, and E; polar and relatively large: R, H, and K; nonpolar and relatively small: I, L, M, and V; nonpolar and relatively large: F, W, and Y. Changes among groups are considered radical whereas changes within groups are considered conservative.

### Estimating the percentage of amino acid mutations in MTBC that are likely to be functional.

The weakened purifying selection in MTBC appears to allow amino acid changes that would be removed by purifying selection in M. canettii and other mycobacteria to persist. In M. canettii the ratio between the number of mutations falling at conserved sites and mutations falling at variable sites is 9/23. Had selection been as strong in MTBC as it was in *M. canettii,* and assuming conservatively that the mutations at the variable sites are entirely neutral, we would expect this ratio to hold for MTBC. Considering that there are 71 mutations at variable sites in MTBC we would expect to see ∼27 mutations at conserved sites within MTBC. Given that, in fact, we find 97 such mutations, we can deduce that, had selection been as strong in MTBC as in *M. canettii,* 70 of the mutations at conserved sites would have been removed. The total number of amino acid mutations observed in our dataset in MTBC is 168. Thus, had selection been as strong as it is in M. canettii we would expect 70/168 (42%) amino acid mutations to have been removed. Since those mutations that would have been removed by selection had it been stronger are likely to have functional consequences, we can deduce that close to 40% of amino acid mutations in MTBC are likely to be functional.

To conservatively estimate the number of synonymous, nonsynonymous and amino acid differences for fully sequenced strains of *M. tuberculosis*, the complete annotated proteomes of the following six fully sequenced MTBC strains were downloaded from the NCBI FTP server (ftp://ftp.ncbi.nlm.nih.gov/): M. tuberculosis H37Rv, M. tuberculosis CDC1551, M. tuberculosis H37Ra, M. tuberculosis F11, M. bovis and *M. bovis* BCS Pasteur 1173P2. In order to locate the clear orthologs of the genes in the H37Rv strain, we conducted a bidirectional FASTA search of each protein sequence from the H37Rv strain of M. tuberculosis in each of the complete proteomes of the five other strains. A M. tuberculosis H37Rv protein was considered to have a clear ortholog in another Mycobacterium if it had a reciprocal best hit and if the synteny was maintained. Specifically, only pairs of genes that were reciprocal best hits and for which both adjacent genes were also each other's reciprocal best hits were considered orthologous. We further eliminated from consideration gene pairs that could not be aligned across their entire protein sequence at the DNA level or that had gaps in their DNA sequence alignment that could indicate frameshifts. We also removed from the analysis genes that were clearly outliers and had over ten amino acid differences between the strain pairs. This left us with 3,231 gene pairs for the H37Rv/F11 comparison, 2,568 gene pairs for the H37Rv/CDC1551 comparison, 3,495 gene pairs for the H37Rv/H37Ra genes, 3,325 gene pairs for the H37Rv/M. bovis comparison and 3,218 gene pairs for the H37Rv/M. bovis BCG comparison. For these protein pairs we counted the number of synonymous and nonsynonymous DNA changes based on their FASTA pairwise DNA-level alignments. For the H37Rv/CDC1551 and H37Rv/F11 comparisons, we also counted the amino acid differences based on their FASTA protein level pairwise alignments.

To calculate the average, minimum, and maximum number of functional differences we expect to see for any given pair of M. tuberculosis strains, we considered the following. We examined our dataset and found that for the total of 89 genes sequenced, the number of pairwise differences between MTBC strains ranged between zero and 46, with an average of 25. If for a fully sequenced pair of strains we could find *X* amino acid differences, and for the same pair of strains we found in our dataset *Y* differences, and given that ∼40% of amino acid differences are expected to be functional, we can extrapolate that the average number of differences expected between any given pair of strains is:


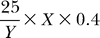


For the two comparisons that could be carried out with the three fully sequenced strains that were also analyzed in our study, this gave between 249 differences (H37Rv/CDC1551 comparison, *X* = 423, *Y* = 17) and 315 differences (H37Rv/F11 comparison, *X* = 410, *Y* = 13).

Similarly, the maximal number of functional differences expected is


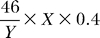


For the two comparisons we could perform this gave between 458 (H37Rv/CDC1551 comparison) and 580 differences (H37Rv/F11 comparison).

The minimal number expected is


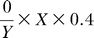


which of course gives zero for both comparisons.

To investigate the correlation between genetic and geographic distances, we computed great-circle geographic distances between all pairs of strains, both for routes that allow crossing of the Earth's major bodies of water (“water routes”), and for those that do not (“land routes”). To do so, we used the haversine method of Ramachandran et al. [59], along with the five waypoints that these authors defined for land route distances. Then, for each type of geographic distance, we computed the coefficient of linear correlation between geographic and genetic distances among strains, and tested its significance using the Mantel test.

## Supporting Information

Figure S1 Position of the 89 Genes Included in this Study Relative to the M. tuberculosis H37Rv Genome Sequence (35 KB DOC)

Figure S2 Neighbor-Joining Phylogeny Using the Concatenated Sequences of 89 Genes in 108 MTBC Strains The distance used is the number of SNPs, and the bootstrap values of 1,000 replicates are indicated for the main branches. Branches are colored according to the lineages defined by our genomic deletion analyses reported previously [19] and correspond to those used in Figure 1. The branch leading to M. canettii has been truncated in the figure because of the large numbers of changes that separate this hypothesized outgroup from the rest of the phylogeny (Table S4). Ancient and modern strain lineages are indicated.
(236 KB DOC)

Figure S3 Human Population Densities in the Year 2000 Western Europe, North India, and China are among the most densely populated areas in the Old World (Source NASA: http://visibleearth.nasa.gov/view_rec.php?id=116).
(2.68 MB DOC)

Table S1 Mycobacterial Strains Included in This Study (185 KB DOC)

Table S2 Genes Sequenced in This Study (197 KB DOC)

Table S3 Primers Used for PCR Amplification and Sequencing (293 KB DOC)

Table S4 SNPs Identified in M. canettii Strain K116 (280 KB DOC)

Table S5 SNPs Identified in 107 MTBC Strains (820 KB DOC)

Table S6 Nonsynonymous and Synonymous SNPs Identified in 107 M. tuberculosis Complex Strains by Gene Category, Phylogenetic Clade, and Branch in the Phylogenetic Tree (Figure 1) (50 KB DOC)

## Abbreviations

MTBC: Mycobacterium tuberculosis complex
SNP: single nucleotide polymorphism
