# Supplementary material for: High Functional Diversity in Mycobacterium tuberculosis Driven by Genetic Drift and Human Demography
Source: PLoS Biol. 2008 Dec 16;6(12):e311. doi: 10.1371/journal.pbio.0060311 (PMC2602723; doi:10.1371/journal.pbio.0060311)
Supplement: Supplementary file 1 [file pbio.0060311.sg001.doc]

**Figure S1**

Position of the 89 genes included in this study relative to the *Mycobacterium tuberculosis* H37Rv genome sequence.
