# Supplementary material for: High Functional Diversity in Mycobacterium tuberculosis Driven by Genetic Drift and Human Demography
Source: PLoS Biol. 2008 Dec 16;6(12):e311. doi: 10.1371/journal.pbio.0060311 (PMC2602723; doi:10.1371/journal.pbio.0060311)
Supplement: Supplementary file 2 [file pbio.0060311.sg002.doc]

**Figure S2**

Neighbor-joining phylogeny based on the concatenated sequences of 89 genes in 108 *M. tuberculosis* complex strains. The distance used is the number of SNPs and the bootstrap values of 1000 replicates are indicated for the main branches. Branches are colored based on the lineages defined by our genomic deletion analyses reported previously [19] and correspond to those used in Figure 1. The branch leading to *M. canettii* has been truncated in the figure because of the large numbers of changes that separate this hypothesized outgroup from the rest of the phylogeny (Table S4).
