# Supplementary material for: High Functional Diversity in Mycobacterium tuberculosis Driven by Genetic Drift and Human Demography
Source: PLoS Biol. 2008 Dec 16;6(12):e311. doi: 10.1371/journal.pbio.0060311 (PMC2602723; doi:10.1371/journal.pbio.0060311)
Supplement: Supplementary file 3 [file pbio.0060311.sg003.doc]

**Figure S3**

Human population densities in the year 2000. Western Europe, North India, and China are among the most densely populated areas in the Old Word (Source NASA: http://visibleearth.nasa.gov/view_rec.php?id=116).
