# Supplementary material for: High Functional Diversity in Mycobacterium tuberculosis Driven by Genetic Drift and Human Demography
Source: PLoS Biol. 2008 Dec 16;6(12):e311. doi: 10.1371/journal.pbio.0060311 (PMC2602723; doi:10.1371/journal.pbio.0060311)
Supplement: Supplementary file 4 [file pbio.0060311.st001.doc]

**Table S1.** Mycobacterial strains included in this study.

| **Strain** | **Traditional Species Designation** | **Main LSP Deleted^1^** | **Origin** | **Comments** |
| --- | --- | --- | --- | --- |
| 00_1695 | *M. tuberculosis* | RD105 | Japan |  |
| 00_2786 | *M. tuberculosis* | RD750 | India |  |
| 01_1007 | *M. tuberculosis* | RD105 | Indonesia |  |
| 01_2382 | *M. tuberculosis* | RD239 | Thailand |  |
| 02_1987 | *M. tuberculosis* | RD105 | South Korea |  |
| 02_3351 | *M. tuberculosis* | RD105 | Mongolia |  |
| 10010_03 | *M. africanum* | RD711 | Sierra Leone |  |
| 11821_03 | *M. africanum* | RD711 | Sierra Leone |  |
| 3494_04 | *M. africanum* | RD711 | Sierra Leone |  |
| 4141_04 | *M. africanum* | RD702 | Sierra Leone |  |
| 4783_04 | *M. tuberculosis* | pks15/1 7bp | Sierra Leone |  |
| 5333_04 | *M. africanum* | RD711 | Ghana |  |
| 5336_04 | *M. africanum* | RD702 | Ghana |  |
| 5415_04 | *M. africanum* | RD702 | Ghana |  |
| 5444_04 | *M. africanum* | RD711 | Ghana |  |
| 5492_04 | *M. tuberculosis* | pks15/1 7bp | Ghana |  |
| 5536_04 | *M. africanum* | RD711 | Ghana |  |
| 91_0079 | *M. tuberculosis* | RD750 | Ethiopia |  |
| 92_0320 | *M. tuberculosis* | RD750 | India |  |
| 93_1117 | *M. tuberculosis* | RD105 | USA |  |
| 94_M4241A | *M. tuberculosis* | RD105 | China |  |
| 95_0545 | *M. tuberculosis* | RD239 | Laos |  |
| 95_1646 | *M. tuberculosis* | RD750 | Pakistan |  |
| 95_1848 | *M. tuberculosis* | RD105 | China |  |
| 96_0982 | *M. tuberculosis* | RD750 | Pakistan |  |
| 96_1548 | *M. tuberculosis* | RD105 | Laos |  |
| 96_2104 | *M. tuberculosis* | RD105 | Vietnam |  |
| 96_4329 | *M. tuberculosis* | RD239 | Burma |  |
| 97_0827 | *M. tuberculosis* | RD105 | Singapore |  |
| 98_1663 | *M. tuberculosis* | RD105 | China |  |
| 98_1833 | *M. tuberculosis* | RD105 | China |  |
| 98_1863 | *M. tuberculosis* | RD105 | China |  |
| 99_2235 | *M. tuberculosis* | RD750 | Vietnam |  |
| 993693b | *M. tuberculosis* | RD750 | Ethiopia |  |
| ATCC35711 | *M. africanum* | RD702 | Senegal |  |
| C11 | *M. tuberculosis* | pks15/1 7bp | South Africa |  |
| C4 | *M. tuberculosis* | pks15/1 7bp | South Africa |  |
| CPHL_A | *M. africanum* | RD711 | South Africa |  |
| EAS009 | *M. tuberculosis* | RD239 | Cambodia |  |
| EAS050 | *M. tuberculosis* | RD239 | Indonesia |  |
| EAS053 | *M. tuberculosis* | RD239 | India |  |
| EAS054 | *M. tuberculosis* | RD239 | India |  |
| Erdman | *M. tuberculosis* | pks15/1 7bp | USA | laboratory strain |
| GM_0981 | *M. africanum* | RD702 | The Gambia |  |
| GM_1121 | *M. africanum* | RD702 | The Gambia |  |
| GM_1255 | *M. africanum* | RD702 | The Gambia |  |
| GM_1503 | *M. tuberculosis* | pks15/1 7bp | The Gambia |  |
| K100 | *M. tuberculosis* | RD105 | East Asia^2^ |  |
| K11 | *M. tuberculosis* | pks15/1 7bp | Greenland |  |
| K19 | *M. tuberculosis* | RD750 | India |  |
| K21 | *M. tuberculosis* | RD239 | Zimbabwe |  |
| K37 | *M. tuberculosis* | pks15/1 7bp | Uganda |  |
| K49 | *M. tuberculosis* | RD750 | Tanzania |  |
| K6 | *M. africanum* | RD702 | West Africa^2^ |  |
| K67 | *M. tuberculosis* | RD239 | Comoro Islands |  |
| K72 | *M. tuberculosis* | pks15/1 7bp | Central African Republic |  |
| K85 | *M. africanum* | RD702 | West Africa^2^ |  |
| K93 | *M. tuberculosis* | RD239 | Tanzania |  |
| LA19 | *M. tuberculosis* | pks15/1 7bp | Nicaragua |  |
| LA4 | *M. tuberculosis* | pks15/1 7bp | Guatemala |  |
| M4100A | *M. tuberculosis* | RD105 | South Korea |  |
| P3 | *M. tuberculosis* | pks15/1 7bp | Peru |  |
| RIVM9400207 | *M. tuberculosis* | RD750 | Somalia |  |
| RIVM9400594 | *M. tuberculosis* | RD750 | Somalia |  |
| SG1 | *M. tuberculosis* | RD750 | India |  |
| SG2 | *M. africanum* | RD702 | The Gambia |  |
| T16 | *M. tuberculosis* | pks15/1 7bp | Puerto Rico |  |
| T17 | *M. tuberculosis* | RD239 | Philippines |  |
| T22 | *M. tuberculosis* | pks15/1 7bp | Colombia |  |
| T25 | *M. tuberculosis* | RD239 | China |  |
| T26 | *M. tuberculosis* | RD105 | Vietnam |  |
| T28 | *M. tuberculosis* | RD105 | Cambodia |  |
| T30 | *M. tuberculosis* | pks15/1 7bp | USA |  |
| T31 | *M. tuberculosis* | RD239 | Philippines |  |
| T36 | *M. tuberculosis* | RD239 | Philippines |  |
| T38 | *M. tuberculosis* | pks15/1 7bp | USA |  |
| T43 | *M. tuberculosis* | pks15/1 7bp | Mexico |  |
| T46 | *M. tuberculosis* | RD239 | Philippines |  |
| T47 | *M. tuberculosis* | RD105 | Macau |  |
| T48 | *M. tuberculosis* | RD239 | Philippines |  |
| T51 | *M. tuberculosis* | pks15/1 7bp | USA |  |
| T60 | *M. tuberculosis* | pks15/1 7bp | USA |  |
| T61 | *M. tuberculosis* | pks15/1 7bp | China |  |
| T67 | *M. tuberculosis* | RD105 | China |  |
| T7 | *M. tuberculosis* | pks15/1 7bp | USA |  |
| T78 | *M. tuberculosis* | pks15/1 7bp | USA |  |
| T80 | *M. tuberculosis* | RD105 | China |  |
| T83 | *M. tuberculosis* | RD239 | Vietnam |  |
| T84 | *M. tuberculosis* | RD105 | USA |  |
| T85 | *M. tuberculosis* | RD105 | China |  |
| T92 | *M. tuberculosis* | RD239 | Philippines |  |
| T97 | *M. tuberculosis* | RD105 | Hong Kong |  |
| T99 | *M. tuberculosis* | pks15/1 7bp | Iran |  |
| H37Rv | *M. tuberculosis* | pks15/1 7bp | USA | laboratory strain, genome sequenced (Sanger Center) |
| CDC1551 | *M. tuberculosis* | pks15/1 7bp | USA | genome sequenced (TIGR) |
| C-strain | *M. tuberculosis* | pks15/1 7bp | USA | genome sequenced (Broad Institute ) |
| F11 | *M. tuberculosis* | pks15/1 7bp | South Africa | genome sequenced (Broad Institute) |
| Haarlem | *M. tuberculosis* | pks15/1 7bp | USA | genome sequenced (Broad Institute) |
| 210 | *M. tuberculosis* | RD105 | USA | genome sequenced (TIGR) |
| K116 | *M. canettii* | none | Somalia | outgroup |
| bovis | *M. bovis* | RD4 | United Kingdom | pathogen of cattle; genome sequenced (Sanger) |
| BCG | derived from *M. bovis* | RD4 | France | vaccine, genome sequenced (Sanger Center) |
| microti | *M. microti* | RD9 | United Kingdom | pathogen of voles, genome sequenced (Sanger Center) |
| K25 | *M. microti* | RD9 | United Kingdom | pathogen of voles |
| 9577_99 | *M. caprae* | RD9 | Germany | pathogen of goats |
| 8986_99 | *M. caprae* | RD9 | Germany | pathogen of goats |
| 751_01 | *M. bovis* | RD4 | Germany | pathogen of cattle |
| 7739_01 | *M. pinnipedii* | RD9 |  | pathogen of seals and sea lions |

^1^ according to our previous work reported in Ref. 19.

^2^ no country-specific information available
