# Supplementary material for: High Functional Diversity in Mycobacterium tuberculosis Driven by Genetic Drift and Human Demography
Source: PLoS Biol. 2008 Dec 16;6(12):e311. doi: 10.1371/journal.pbio.0060311 (PMC2602723; doi:10.1371/journal.pbio.0060311)
Supplement: Supplementary file 5 [file pbio.0060311.st002.doc]

**Table S2.** Genes sequenced in this study.

| **Rv Nr.** | **Gene Name** | **Gene Category** | **Size (bp)** | **MTBC sSNPs** | **MTBC nSNPs** | ***M. canettii* sSNPs** | ***M. canettii* nSNPs** | **# of distant Mycobacteria in which ortholog could be found** |
| --- | --- | --- | --- | --- | --- | --- | --- | --- |
| 0005 | gyrB | housekeeping | 2145 | 9 | 6 | 3 | 0 | 10 |
| 0006 | gyrA | housekeeping | 2517 | 12 | 16 | 22 | 1 | 10 |
| 0009 | cfp22 | surface/secreted | 549 | 4 | 1 | 3 | 0 | 10 |
| 0040c | mtc28 | surface/secreted | 933 | 3 | 4 | 3 | 2 | 10 |
| 0079 |  | virulence/hypoxia | 822 | 1 | 4 | 0 | 1 | 7 |
| 0129c | fbpC | surface/secreted | 1023 | 1 | 2 | 0 | 0 | 10 |
| 0164 | TB18.5 | surface/secreted | 486 | 1 | 2 | 0 | 1 | 10 |
| 0251c | hsp | virulence/hypoxia | 480 | 0 | 4 | 1 | 1 | 9 |
| 0287 | esxG | surface/secreted | 294 | 0 | 2 | 1 | 0 | 10 |
| 0288 | esxH | surface/secreted | 291 | 0 | 6 | 0 | 0 | 7 |
| 0407 | fgd1 | housekeeping | 1011 | 2 | 3 | 2 | 2 | 10 |
| 0410c | pknG | housekeeping | 2253 | 4 | 8 | 0 | 0 | 10 |
| 413 | mutT3 | housekeeping | 654 | 2 | 3 | 0 | 0 | 3 |
| 0429c | def | housekeeping | 594 | 1 | 1 | 1 | 0 | 10 |
| 0467 | icl1 | housekeeping | 1287 | 5 | 3 | 0 | 0 | 9 |
| 0475 | hbhA | surface/secreted | 600 | 2 | 2 | 1 | 0 | 10 |
| 0569 |  | surface/secreted | 267 | 0 | 1 | 0 | 0 | 3 |
| 0652 | rplL | housekeeping | 393 | 0 | 2 | 0 | 0 | 10 |
| 0798c | cfp29 | surface/secreted | 798 | 2 | 1 | 0 | 0 | 6 |
| 0909 |  | surface/secreted | 180 | 0 | 1 | 0 | 0 | 9 |
| 0934 | pstS1 | surface/secreted | 1125 | 0 | 3 | 1 | 0 | 0 |
| 1009 | rpfB | surface/secreted | 1089 | 2 | 7 | 1 | 2 | 10 |
| 1072 |  | surface/secreted | 837 | 0 | 2 | 0 | 0 | 10 |
| 1160 | mutT2 | housekeeping | 426 | 0 | 2 | 1 | 1 | 9 |
| 1173 | fbiC | housekeeping | 2571 | 0 | 3 | 2 | 0 | 10 |
| 1174c | TB8.4 | surface/secreted | 333 | 0 | 1 | 0 | 0 | 8 |
| 1184c |  | surface/secreted | 1080 | 2 | 1 | 0 | 0 | 9 |
| 1195 | PE13 | surface/secreted | 300 | 0 | 0 | 1 | 0 | 0 |
| 1305 | atpE | housekeeping | 246 | 2 | 1 | 0 | 0 | 10 |
| 1316c | ogt | housekeeping | 498 | 1 | 2 | 0 | 0 | 10 |
| 1411c | lprG | surface/secreted | 711 | 2 | 1 | 0 | 1 | 10 |
| 1636 | TB15.3 | surface/secreted | 441 | 0 | 1 | 0 | 0 | 10 |
| 1733c |  | surface/secreted | 633 | 2 | 3 | 1 | 3 | 7 |
| 1738 |  | virulence/hypoxia | 285 | 0 | 0 | 0 | 0 | 2 |
| 1813c |  | virulence/hypoxia | 432 | 2 | 1 | 0 | 0 | 0 |
| 1827 | cfp17 | surface/secreted | 489 | 0 | 0 | 0 | 0 | 10 |
| 1884c | rpfC | surface/secreted | 531 | 0 | 1 | 0 | 1 | 2 |
| 1886c | fbpB | surface/secreted | 978 | 2 | 4 | 0 | 0 | 10 |
| 1926c | mpt63 | surface/secreted | 480 | 0 | 2 | 0 | 0 | 9 |
| 1980c | mpt64 | surface/secreted | 687 | 2 | 1 | 0 | 1 | 1 |
| 1984c | cfp21 | surface/secreted | 654 | 0 | 1 | 0 | 0 | 2 |
| 1996 |  | virulence/hypoxia | 954 | 2 | 3 | 1 | 0 | 1 |
| 2007c | fdxA | virulence/hypoxia | 345 | 0 | 1 | 0 | 0 | 9 |
| 2030c |  | virulence/hypoxia | 2046 | 4 | 4 | 0 | 1 | 5 |
| 2031c | hspX | surface/secreted | 435 | 2 | 0 | 0 | 0 | 4 |
| 2032 | acg | virulence/hypoxia | 996 | 3 | 5 | 2 | 0 | 8 |
| 2185c | TB16.3 | surface/secreted | 435 | 3 | 1 | 1 | 0 | 10 |
| 2330c | lppP | surface/secreted | 528 | 1 | 3 | 0 | 0 | 9 |
| 2389c | rpfD | surface/secreted | 465 | 0 | 0 | 0 | 0 | 0 |
| 2450c | rpfE | surface/secreted | 519 | 2 | 4 | 2 | 0 | 2 |
| 2462c | tig | surface/secreted | 1401 | 3 | 4 | 0 | 0 | 10 |
| 2626c |  | surface/secreted | 432 | 3 | 0 | 0 | 0 | 1 |
| 2627c |  | virulence/hypoxia | 1242 | 2 | 6 | 1 | 3 | 5 |
| 2628 |  | virulence/hypoxia | 363 | 1 | 7 | 2 | 2 | 0 |
| 2686c |  | surface/secreted | 759 | 3 | 0 | 11 | 6 | 6 |
| 2687c |  | surface/secreted | 714 | 1 | 5 | 4 | 1 | 6 |
| 2744c | 35kd_ag | virulence/hypoxia | 813 | 2 | 6 | 0 | 0 | 9 |
| 2873 | mpt83 | surface/secreted | 663 | 1 | 1 | 0 | 1 | 5 |
| 2875 | mpt70 | surface/secreted | 582 | 2 | 2 | 1 | 0 | 1 |
| 2924c | fpg | housekeeping | 870 | 1 | 6 | 0 | 0 | 10 |
| 2949c |  | housekeeping | 600 | 2 | 3 | 1 | 0 | 2 |
| 2952 |  | housekeeping | 813 | 0 | 4 | 1 | 1 | 3 |
| 2957 |  | housekeeping | 828 | 2 | 5 | 0 | 0 | 0 |
| 2958c |  | housekeeping | 1287 | 1 | 3 | 0 | 0 | 0 |
| 2959c |  | housekeeping | 738 | 3 | 6 | 1 | 0 | 1 |
| 2962c |  | housekeeping | 1350 | 3 | 6 | 1 | 0 | 2 |
| 2985 | mutT1 | housekeeping | 954 | 1 | 1 | 0 | 0 | 10 |
| 3126c |  | virulence/hypoxia | 315 | 0 | 0 | 0 | 0 | 0 |
| 3129 |  | virulence/hypoxia | 333 | 0 | 1 | 0 | 2 | 9 |
| 3132c | devS | virulence/hypoxia | 1737 | 5 | 3 | 1 | 1 | 1 |
| 3133c | dosR | virulence/hypoxia | 654 | 1 | 1 | 0 | 0 | 9 |
| 3221c | TB7.3 | surface/secreted | 216 | 1 | 2 | 0 | 0 | 10 |
| 3261 | fbiA | housekeeping | 996 | 2 | 2 | 0 | 0 | 10 |
| 3262 | fbiB | housekeeping | 1347 | 2 | 1 | 0 | 0 | 10 |
| 3354 | TB9.7 | surface/secreted | 390 | 1 | 1 | 0 | 1 | 10 |
| 3407 |  | surface/secreted | 300 | 0 | 1 | 1 | 0 | 1 |
| 3418c | groES | surface/secreted | 303 | 0 | 0 | 1 | 0 | 10 |
| 3547 |  | housekeeping | 456 | 1 | 2 | 3 | 0 | 9 |
| 3589 | mutY | housekeeping | 915 | 1 | 2 | 0 | 1 | 10 |
| 3763 | lpqH | surface/secreted | 480 | 1 | 2 | 1 | 0 | 10 |
| 3804c | fbpA | surface/secreted | 1017 | 3 | 2 | 0 | 0 | 8 |
| 3872 | PE35 | surface/secreted | 300 | 0 | 2 | 0 | 1 | 6 |
| 3874 | esxB | surface/secreted | 303 | 1 | 1 | 0 | 0 | 7 |
| 3875 | esxA | surface/secreted | 288 | 1 | 3 | 0 | 0 | 7 |
| 3890c | esxC | surface/secreted | 288 | 0 | 2 | 0 | 0 | 3 |
| 3891c | esxD | surface/secreted | 324 | 1 | 1 | 0 | 0 | 2 |
| 3904c | esxE | surface/secreted | 273 | 0 | 2 | 0 | 0 | 8 |
| 3905c | esxF | surface/secreted | 312 | 2 | 2 | 0 | 0 | 7 |
| 3908 | mut4 | housekeeping | 747 | 2 | 3 | 0 | 0 | 10 |
| **Total** |  |  | **65829** | **139** | **231** | **80** | **38** |  |
