# Supplementary material for: High Functional Diversity in Mycobacterium tuberculosis Driven by Genetic Drift and Human Demography
Source: PLoS Biol. 2008 Dec 16;6(12):e311. doi: 10.1371/journal.pbio.0060311 (PMC2602723; doi:10.1371/journal.pbio.0060311)
Supplement: Supplementary file 6 [file pbio.0060311.st003.doc]

**Table S3.** Primers used for PCR amplification and sequencing.

| **Rv Nr.** | **PCR Start** | **PCR Stop** | **Product Size** | **Forward PCR Primer / Internal Sequencing Primer(s)** | **Reverse PCR Primer** |
| --- | --- | --- | --- | --- | --- |
| Rv0005/Rv0006_A | [4979](http://genolist.pasteur.fr/TubercuList/genome.cgi?gene_list_from+from=4.979+to=+location=kb+chkOrg0=2+TotOrg=1) | [6453](http://genolist.pasteur.fr/TubercuList/genome.cgi?gene_list_from+from=6.453+to=+location=kb+chkOrg0=2+TotOrg=1) | 1474 | GCGCGACACCTACGGATAACACG | ACTTTCGCGTCGGTGGGGTTGG |
|  |  |  |  | 1) CTGCTTGAGGATGGCGGTGTC |  |
| Rv0005/Rv0006_B | [6205](http://genolist.pasteur.fr/TubercuList/genome.cgi?gene_list_from+from=6.205+to=+location=kb+chkOrg0=2+TotOrg=1) | [7681](http://genolist.pasteur.fr/TubercuList/genome.cgi?gene_list_from+from=7.681+to=+location=kb+chkOrg0=2+TotOrg=1) | 1476 | CGCGCTGACGTCGGTGGTGAAC | ATCGCCGCCGGTGGGTCATTG |
|  |  |  |  | 1) CGCGCAAGTCCGAACTGTATG |  |
| Rv0005/Rv0006_C | [7456](http://genolist.pasteur.fr/TubercuList/genome.cgi?gene_list_from+from=7.456+to=+location=kb+chkOrg0=2+TotOrg=1) | [8918](http://genolist.pasteur.fr/TubercuList/genome.cgi?gene_list_from+from=8.918+to=+location=kb+chkOrg0=2+TotOrg=1) | 1462 | ATCGCCGGGTGCTCTATG | GCGTTTCTGGCTGCGATAC |
|  |  |  |  | 1) TCGGCATGGCAACCAATATCC |  |
| Rv0005/Rv0006_D | [8625](http://genolist.pasteur.fr/TubercuList/genome.cgi?gene_list_from+from=8.625+to=+location=kb+chkOrg0=2+TotOrg=1) | [10143](http://genolist.pasteur.fr/TubercuList/genome.cgi?gene_list_from+from=10.143+to=+location=kb+chkOrg0=2+TotOrg=1) | 1518 | CGCCTGGCCGCACTGGAACG | GCGGCGGGCGGGTTGGTC |
|  |  |  |  | 1) GAGGAACGCATCGCCCAGGTC |  |
| Rv0009 | [12308](http://genolist.pasteur.fr/TubercuList/genome.cgi?gene_list_from+from=12.308+to=+location=kb+chkOrg0=2+TotOrg=1) | [13099](http://genolist.pasteur.fr/TubercuList/genome.cgi?gene_list_from+from=13.099+to=+location=kb+chkOrg0=2+TotOrg=1) | 791 | TCATTTTGCCGCCTTTCTGTGTCC | CCCCAACGGCGTGTCGGTGTA |
| Rv0040c | [42307](http://genolist.pasteur.fr/TubercuList/genome.cgi?gene_list_from+from=42.307+to=+location=kb+chkOrg0=2+TotOrg=1) | [43573](http://genolist.pasteur.fr/TubercuList/genome.cgi?gene_list_from+from=43.573+to=+location=kb+chkOrg0=2+TotOrg=1) | 1266 | GGACGCCGCCGCCGCACAC | CGATTCGGTCACGCCCAACAG |
|  |  |  |  | 1) CAGCAACAGGTTCTCACCGGC |  |
| Rv0079 | [88127](http://genolist.pasteur.fr/TubercuList/genome.cgi?gene_list_from+from=88.127+to=+location=kb+chkOrg0=2+TotOrg=1) | [89107](http://genolist.pasteur.fr/TubercuList/genome.cgi?gene_list_from+from=89.107+to=+location=kb+chkOrg0=2+TotOrg=1) | 980 | GGCCCTGTCCGCGTCCGTGTCG | GCCAGCAACCGCATCGCCTCGTCA |
| Rv0129c | [156457](http://genolist.pasteur.fr/TubercuList/genome.cgi?gene_list_from+from=156.457+to=+location=kb+chkOrg0=2+TotOrg=1) | [157707](http://genolist.pasteur.fr/TubercuList/genome.cgi?gene_list_from+from=157.707+to=+location=kb+chkOrg0=2+TotOrg=1) | 1250 | AGTGGCGCTGGCGAAGGGGTGAC | GCGCTGCGGCCACGACATTC |
| Rv0164 | [193528](http://genolist.pasteur.fr/TubercuList/genome.cgi?gene_list_from+from=193.528+to=+location=kb+chkOrg0=2+TotOrg=1) | [194217](http://genolist.pasteur.fr/TubercuList/genome.cgi?gene_list_from+from=194.217+to=+location=kb+chkOrg0=2+TotOrg=1) | 689 | GAGGCCATTCGGTCGCTGTTGTCG | ACAGCCGCCGTAATCGAGCACACC |
| Rv0251c | [301964](http://genolist.pasteur.fr/TubercuList/genome.cgi?gene_list_from+from=301.964+to=+location=kb+chkOrg0=2+TotOrg=1) | [302889](http://genolist.pasteur.fr/TubercuList/genome.cgi?gene_list_from+from=302.889+to=+location=kb+chkOrg0=2+TotOrg=1) | 925 | GTCACGCACCGCCGCAGTCC | GCGCGAACTCCCAGCCGTAGG |
| Rv0287/Rv0288 | [351437](http://genolist.pasteur.fr/TubercuList/genome.cgi?gene_list_from+from=351.437+to=+location=kb+chkOrg0=2+TotOrg=1) | [352224](http://genolist.pasteur.fr/TubercuList/genome.cgi?gene_list_from+from=352.224+to=+location=kb+chkOrg0=2+TotOrg=1) | 787 | GAGAGGGGGAGGCGACGGCTTACC | TCCCCGCCCCAATGGTTTCAGC |
| Rv0407 | [490594](http://genolist.pasteur.fr/TubercuList/genome.cgi?gene_list_from+from=490.594+to=+location=kb+chkOrg0=2+TotOrg=1) | [492199](http://genolist.pasteur.fr/TubercuList/genome.cgi?gene_list_from+from=492.199+to=+location=kb+chkOrg0=2+TotOrg=1) | 1605 | CGTGGCCGCGAGCGAGGTGAA | CGCCCGAACCGTCAACAACACTGG |
|  |  |  |  | 1) CGGAGTTCAAGGAGCGGTTCG |  |
| Rv0410c | [494922](http://genolist.pasteur.fr/TubercuList/genome.cgi?gene_list_from+from=494.922+to=+location=kb+chkOrg0=2+TotOrg=1) | [497541](http://genolist.pasteur.fr/TubercuList/genome.cgi?gene_list_from+from=497.541+to=+location=kb+chkOrg0=2+TotOrg=1) | 2619 | CCGATCGCCGTGCTGGTGGTT | CTGGCCGGGCTGGTAGAGGAAGAC |
|  |  |  |  | 1) TGAGGCCCTGATGACCAACCC |  |
|  |  |  |  | 2) GGCGCTGAGCTACCTGCATTC |  |
|  |  |  |  | 3) GCGCACACCGACGTGTATCTG |  |
| Rv0413 | [499641](http://genolist.pasteur.fr/TubercuList/genome.cgi?gene_list_from+from=499.641+to=+location=kb+chkOrg0=2+TotOrg=1) | [500556](http://genolist.pasteur.fr/TubercuList/genome.cgi?gene_list_from+from=500.556+to=+location=kb+chkOrg0=2+TotOrg=1) | 915 | CGGCTAACGCAACGAAACTCT | CCGGGTCTGGGGCTGGTA |
| Rv0429c | [517565](http://genolist.pasteur.fr/TubercuList/genome.cgi?gene_list_from+from=517.565+to=+location=kb+chkOrg0=2+TotOrg=1) | [518469](http://genolist.pasteur.fr/TubercuList/genome.cgi?gene_list_from+from=518.469+to=+location=kb+chkOrg0=2+TotOrg=1) | 904 | GCGGCGGCGTGTTCAAGTGC | CTAAGCCGATTGCCCGCCTCCTCA |
| Rv0467 | [557283](http://genolist.pasteur.fr/TubercuList/genome.cgi?gene_list_from+from=557.283+to=+location=kb+chkOrg0=2+TotOrg=1) | [558908](http://genolist.pasteur.fr/TubercuList/genome.cgi?gene_list_from+from=558.908+to=+location=kb+chkOrg0=2+TotOrg=1) | 1625 | TACCTGCGGATTTGTCGTTACTGG | ATCGCATCGCTCACTGTTCCTC |
|  |  |  |  | 1) CGCGCTGACCGGCAACATGGC |  |
|  |  |  |  | 2) CAGCAGCACATCCGCACTTTG |  |
| Rv0475 | [565606](http://genolist.pasteur.fr/TubercuList/genome.cgi?gene_list_from+from=565.606+to=+location=kb+chkOrg0=2+TotOrg=1) | [566639](http://genolist.pasteur.fr/TubercuList/genome.cgi?gene_list_from+from=566.639+to=+location=kb+chkOrg0=2+TotOrg=1) | 1033 | GCTGGGCCTGACATCTACGAACTC | CGCGCCCAGGATCACCAACC |
| Rv0569 | [660836](http://genolist.pasteur.fr/TubercuList/genome.cgi?gene_list_from+from=660.836+to=+location=kb+chkOrg0=2+TotOrg=1) | [661348](http://genolist.pasteur.fr/TubercuList/genome.cgi?gene_list_from+from=661.348+to=+location=kb+chkOrg0=2+TotOrg=1) | 512 | GGCGGGCCGTCCAGAGC | CGGCACCAGCGTTCCATCC |
| Rv0652 | [748711](http://genolist.pasteur.fr/TubercuList/genome.cgi?gene_list_from+from=748.711+to=+location=kb+chkOrg0=2+TotOrg=1) | [749439](http://genolist.pasteur.fr/TubercuList/genome.cgi?gene_list_from+from=749.439+to=+location=kb+chkOrg0=2+TotOrg=1) | 728 | AAGGCGGCCGGGTTGTTCA | CCGCGGCGCAGTTTTGGTC |
| Rv0798c | [891361](http://genolist.pasteur.fr/TubercuList/genome.cgi?gene_list_from+from=891.361+to=+location=kb+chkOrg0=2+TotOrg=1) | [892395](http://genolist.pasteur.fr/TubercuList/genome.cgi?gene_list_from+from=892.395+to=+location=kb+chkOrg0=2+TotOrg=1) | 1034 | GCCGCCTAGCCCGCACCGTCTTC | GACCGGCGGACTGTTCTTCTCACC |
| Rv0909 | [1014587](http://genolist.pasteur.fr/TubercuList/genome.cgi?gene_list_from+from=1014.587+to=+location=kb+chkOrg0=2+TotOrg=1) | [1015079](http://genolist.pasteur.fr/TubercuList/genome.cgi?gene_list_from+from=1015.079+to=+location=kb+chkOrg0=2+TotOrg=1) | 492 | CCGGCTCTAAGGGCTGGTTGC | TGTACCGCACGATCGTCCACTTGA |
| Rv0934 | [1041930](http://genolist.pasteur.fr/TubercuList/genome.cgi?gene_list_from+from=1041.93+to=+location=kb+chkOrg0=2+TotOrg=1) | [1043370](http://genolist.pasteur.fr/TubercuList/genome.cgi?gene_list_from+from=1043.37+to=+location=kb+chkOrg0=2+TotOrg=1) | 1440 | GCCCAGGCCGCCCGCATCA | CACCGCACCTATCCCACCCAACCA |
| Rv1009 | [1127889](http://genolist.pasteur.fr/TubercuList/genome.cgi?gene_list_from+from=1127.889+to=+location=kb+chkOrg0=2+TotOrg=1) | [1129347](http://genolist.pasteur.fr/TubercuList/genome.cgi?gene_list_from+from=1129.347+to=+location=kb+chkOrg0=2+TotOrg=1) | 1458 | CCGAGCGGCCCATAACACC | CCGGCCCGACCTCCAAAACCA |
| Rv1072 | [1196154](http://genolist.pasteur.fr/TubercuList/genome.cgi?gene_list_from+from=1196.154+to=+location=kb+chkOrg0=2+TotOrg=1) | [1197243](http://genolist.pasteur.fr/TubercuList/genome.cgi?gene_list_from+from=1197.243+to=+location=kb+chkOrg0=2+TotOrg=1) | 1089 | CCGCGGGGGCTAAGGTTT | GCTGCGCCCCCATCTCC |
| Rv1160 | [1286457](http://genolist.pasteur.fr/TubercuList/genome.cgi?gene_list_from+from=1286.457+to=+location=kb+chkOrg0=2+TotOrg=1) | [1287136](http://genolist.pasteur.fr/TubercuList/genome.cgi?gene_list_from+from=1287.136+to=+location=kb+chkOrg0=2+TotOrg=1) | 679 | CGGCCATAAACGTCGGAAACTTGA | GCCACGCGTCCAGAAAACCATC |
| Rv1173_A | [1302828](http://genolist.pasteur.fr/TubercuList/genome.cgi?gene_list_from+from=1302.828+to=+location=kb+chkOrg0=2+TotOrg=1) | [1304314](http://genolist.pasteur.fr/TubercuList/genome.cgi?gene_list_from+from=1304.314+to=+location=kb+chkOrg0=2+TotOrg=1) | 1486 | GCTCGGCCTGGCGCTACTGTTG | CTGCGGTATTGCGGCCCTGAGTGT |
|  |  |  |  | 1) CGCCCAAGGTTCCAGCACGTA |  |
| Rv1173_B | [1304102](http://genolist.pasteur.fr/TubercuList/genome.cgi?gene_list_from+from=1304.102+to=+location=kb+chkOrg0=2+TotOrg=1) | [1305692](http://genolist.pasteur.fr/TubercuList/genome.cgi?gene_list_from+from=1305.692+to=+location=kb+chkOrg0=2+TotOrg=1) | 1590 | GGCTGACCGCGCAACCCAAATA | GCCGGCTCCTGCAACAACTAT |
|  |  |  |  | 1) CGGCGACGAGGTGACCTTTGT |  |
| Rv1174c | [1305463](http://genolist.pasteur.fr/TubercuList/genome.cgi?gene_list_from+from=1305.463+to=+location=kb+chkOrg0=2+TotOrg=1) | [1306218](http://genolist.pasteur.fr/TubercuList/genome.cgi?gene_list_from+from=1306.218+to=+location=kb+chkOrg0=2+TotOrg=1) | 755 | CGCCAGCGCACTACCACATAC | TTGCGGCCAGGCTATAAACTCATT |
| Rv1184c | [1324438](http://genolist.pasteur.fr/TubercuList/genome.cgi?gene_list_from+from=1324.438+to=+location=kb+chkOrg0=2+TotOrg=1) | [1325716](http://genolist.pasteur.fr/TubercuList/genome.cgi?gene_list_from+from=1325.716+to=+location=kb+chkOrg0=2+TotOrg=1) | 1278 | GTGCGCACCCGAAGAAGG | CCGGGACGTGGCTGACTAAGAT |
| Rv1195 | [1338792](http://genolist.pasteur.fr/TubercuList/genome.cgi?gene_list_from+from=1338.792+to=+location=kb+chkOrg0=2+TotOrg=1) | [1339478](http://genolist.pasteur.fr/TubercuList/genome.cgi?gene_list_from+from=1339.478+to=+location=kb+chkOrg0=2+TotOrg=1) | 686 | CTTCGGCGGCGGCGGTAGG | ACGCGGCCGAAAACAGGTCACTC |
| Rv1305 | [1460821](http://genolist.pasteur.fr/TubercuList/genome.cgi?gene_list_from+from=1460.821+to=+location=kb+chkOrg0=2+TotOrg=1) | [1461382](http://genolist.pasteur.fr/TubercuList/genome.cgi?gene_list_from+from=1461.382+to=+location=kb+chkOrg0=2+TotOrg=1) | 561 | CCGGCGGCATTCTGGTC | GACTCGCCGCCTTCCTCTG |
| Rv1316c | [1476954](http://genolist.pasteur.fr/TubercuList/genome.cgi?gene_list_from+from=1476.954+to=+location=kb+chkOrg0=2+TotOrg=1) | [1477770](http://genolist.pasteur.fr/TubercuList/genome.cgi?gene_list_from+from=1477.77+to=+location=kb+chkOrg0=2+TotOrg=1) | 816 | AAGCCTGCCAGCGCCGATGATACT | AGCTGGGCCTGCCTGCACAACGAC |
| Rv1441c | [1618757](http://genolist.pasteur.fr/TubercuList/genome.cgi?gene_list_from+from=1618.757+to=+location=kb+chkOrg0=2+TotOrg=1) | [1619867](http://genolist.pasteur.fr/TubercuList/genome.cgi?gene_list_from+from=1619.867+to=+location=kb+chkOrg0=2+TotOrg=1) | 1110 | TGCCGCCGTCGCCACCTT | GCCACGGCCGCAATGTCG |
| Rv1636 | [1842217](http://genolist.pasteur.fr/TubercuList/genome.cgi?gene_list_from+from=1842.217+to=+location=kb+chkOrg0=2+TotOrg=1) | [1843050](http://genolist.pasteur.fr/TubercuList/genome.cgi?gene_list_from+from=1843.05+to=+location=kb+chkOrg0=2+TotOrg=1) | 833 | GGTGCGCCGGGACGACTC | CCTGCCGACTTCACCCAACTGCTC |
| Rv1733c | [1959647](http://genolist.pasteur.fr/TubercuList/genome.cgi?gene_list_from+from=1959.647+to=+location=kb+chkOrg0=2+TotOrg=1) | [1960562](http://genolist.pasteur.fr/TubercuList/genome.cgi?gene_list_from+from=1960.562+to=+location=kb+chkOrg0=2+TotOrg=1) | 915 | ACGGGCAGTGGTTACAGATGAAGG | GCGCCCAGCCATTCGTGA |
| Rv1738 | [1965554](http://genolist.pasteur.fr/TubercuList/genome.cgi?gene_list_from+from=1965.554+to=+location=kb+chkOrg0=2+TotOrg=1) | [1966044](http://genolist.pasteur.fr/TubercuList/genome.cgi?gene_list_from+from=1966.044+to=+location=kb+chkOrg0=2+TotOrg=1) | 490 | GGCGCGGTGGTCGGATTTGTC | CTCAGGGCCGCCAGTCTTCTCG |
| Rv1813c | [2055541](http://genolist.pasteur.fr/TubercuList/genome.cgi?gene_list_from+from=2055.541+to=+location=kb+chkOrg0=2+TotOrg=1) | [2056242](http://genolist.pasteur.fr/TubercuList/genome.cgi?gene_list_from+from=2056.242+to=+location=kb+chkOrg0=2+TotOrg=1) | 701 | AGCCGGGGCAGCCAGGGTAGG | AGGGCGCCGGGCTTCCAACC |
| Rv1827 | [2072508](http://genolist.pasteur.fr/TubercuList/genome.cgi?gene_list_from+from=2072.508+to=+location=kb+chkOrg0=2+TotOrg=1) | [2073216](http://genolist.pasteur.fr/TubercuList/genome.cgi?gene_list_from+from=2073.216+to=+location=kb+chkOrg0=2+TotOrg=1) | 708 | AAGGGCGATAATAGGCGGTAAAGA | GCCGGGGCGTCACCAGA |
| Rv1884c | [2133641](http://genolist.pasteur.fr/TubercuList/genome.cgi?gene_list_from+from=2133.641+to=+location=kb+chkOrg0=2+TotOrg=1) | [2134329](http://genolist.pasteur.fr/TubercuList/genome.cgi?gene_list_from+from=2134.329+to=+location=kb+chkOrg0=2+TotOrg=1) | 688 | GCGCTGCCATGTGAACTGTG | CAACGGGCCCTGACGACA |
| Rv1886c | [2134677](http://genolist.pasteur.fr/TubercuList/genome.cgi?gene_list_from+from=2134.677+to=+location=kb+chkOrg0=2+TotOrg=1) | [2136032](http://genolist.pasteur.fr/TubercuList/genome.cgi?gene_list_from+from=2136.032+to=+location=kb+chkOrg0=2+TotOrg=1) | 1355 | GCAGCTGAGCACGCCACTTGA | CATACCGCCATACCGTTTGTGAGC |
|  |  |  |  | 1) ACTCGGCTAACTGGCTGGTGC |  |
| Rv1926c | [2178865](http://genolist.pasteur.fr/TubercuList/genome.cgi?gene_list_from+from=2178.865+to=+location=kb+chkOrg0=2+TotOrg=1) | [2179557](http://genolist.pasteur.fr/TubercuList/genome.cgi?gene_list_from+from=2179.557+to=+location=kb+chkOrg0=2+TotOrg=1) | 692 | CGCCATTCCACGCACCACCAG | GTCTGCCCCACGGTTCCTATCG |
| Rv1980c | [2223274](http://genolist.pasteur.fr/TubercuList/genome.cgi?gene_list_from+from=2223.274+to=+location=kb+chkOrg0=2+TotOrg=1) | [2224131](http://genolist.pasteur.fr/TubercuList/genome.cgi?gene_list_from+from=2224.131+to=+location=kb+chkOrg0=2+TotOrg=1) | 857 | TGGGCGCACCGAACACTC | CAGCGCGATGCCCTATGT |
| Rv1984c | [2227795](http://genolist.pasteur.fr/TubercuList/genome.cgi?gene_list_from+from=2227.795+to=+location=kb+chkOrg0=2+TotOrg=1) | [2228707](http://genolist.pasteur.fr/TubercuList/genome.cgi?gene_list_from+from=2228.707+to=+location=kb+chkOrg0=2+TotOrg=1) | 912 | TACCGCCCGACCGTCATTTCATCC | GGCTTGACGCGGACGACCACAGA |
| Rv1996 | [2238896](http://genolist.pasteur.fr/TubercuList/genome.cgi?gene_list_from+from=2238.896+to=+location=kb+chkOrg0=2+TotOrg=1) | [2240089](http://genolist.pasteur.fr/TubercuList/genome.cgi?gene_list_from+from=2240.089+to=+location=kb+chkOrg0=2+TotOrg=1) | 1193 | CACCCCCAGCTAAAGACACTAATG | ATCAGGGCCGTAAGTCATCCA |
| Rv2007c | [2255874](http://genolist.pasteur.fr/TubercuList/genome.cgi?gene_list_from+from=2255.874+to=+location=kb+chkOrg0=2+TotOrg=1) | [2256537](http://genolist.pasteur.fr/TubercuList/genome.cgi?gene_list_from+from=2256.537+to=+location=kb+chkOrg0=2+TotOrg=1) | 663 | CGCCACATTGACCGCAGAAAGT | CCGCGTCGACGTGCCAGTGA |
| Rv2030c_A | [2276208](http://genolist.pasteur.fr/TubercuList/genome.cgi?gene_list_from+from=2276.208+to=+location=kb+chkOrg0=2+TotOrg=1) | [2277634](http://genolist.pasteur.fr/TubercuList/genome.cgi?gene_list_from+from=2277.634+to=+location=kb+chkOrg0=2+TotOrg=1) | 1426 | CGCCGGCCGGGAACAGTGCT | GCCCGGGCCGCCATGACACAGT |
|  |  |  |  | 1) TGATTCGCGGCTTGCCTTCGT |  |
| Rv2030c_B | [2277340](http://genolist.pasteur.fr/TubercuList/genome.cgi?gene_list_from+from=2277.34+to=+location=kb+chkOrg0=2+TotOrg=1) | [2278626](http://genolist.pasteur.fr/TubercuList/genome.cgi?gene_list_from+from=2278.626+to=+location=kb+chkOrg0=2+TotOrg=1) | 1286 | TCGACCGATGCAGGCTGTAAAGAT | TCGCTGCCGGTAGGTGCTGAC |
|  |  |  |  | 1) GTCGGTGGCGGTTTCGGAAGG |  |
| Rv2031c | [2278418](http://genolist.pasteur.fr/TubercuList/genome.cgi?gene_list_from+from=2278.418+to=+location=kb+chkOrg0=2+TotOrg=1) | [2279042](http://genolist.pasteur.fr/TubercuList/genome.cgi?gene_list_from+from=2279.042+to=+location=kb+chkOrg0=2+TotOrg=1) | 624 | CTCGCGGCGGTCACGGAACACG | CGACGGGCGCGGACAAATG |
| Rv2032 | [2278904](http://genolist.pasteur.fr/TubercuList/genome.cgi?gene_list_from+from=2278.904+to=+location=kb+chkOrg0=2+TotOrg=1) | [2280262](http://genolist.pasteur.fr/TubercuList/genome.cgi?gene_list_from+from=2280.262+to=+location=kb+chkOrg0=2+TotOrg=1) | 1358 | TGGCGCTGAACGGGAAGGGTGGTG | CTCAGCCGGCCGGGGACGAATAGC |
| Rv2185c | [2446958](http://genolist.pasteur.fr/TubercuList/genome.cgi?gene_list_from+from=2446.958+to=+location=kb+chkOrg0=2+TotOrg=1) | [2447690](http://genolist.pasteur.fr/TubercuList/genome.cgi?gene_list_from+from=2447.69+to=+location=kb+chkOrg0=2+TotOrg=1) | 732 | CGGCCCCGGCATCACATACC | GACGGGTCGCGGGCAAAAA |
| Rv2330c | [2603538](http://genolist.pasteur.fr/TubercuList/genome.cgi?gene_list_from+from=2603.538+to=+location=kb+chkOrg0=2+TotOrg=1) | [2604301](http://genolist.pasteur.fr/TubercuList/genome.cgi?gene_list_from+from=2604.301+to=+location=kb+chkOrg0=2+TotOrg=1) | 763 | TCCTGGCAGCCTGAAACTCC | GGCACCCGCGCTTTAGGA |
| Rv2389c | [2683046](http://genolist.pasteur.fr/TubercuList/genome.cgi?gene_list_from+from=2683.046+to=+location=kb+chkOrg0=2+TotOrg=1) | [2683881](http://genolist.pasteur.fr/TubercuList/genome.cgi?gene_list_from+from=2683.881+to=+location=kb+chkOrg0=2+TotOrg=1) | 835 | CCAGCTGGGCCGGGGTGTAGGTA | TGGCGGCGTCGGTCAAATCAGC |
| Rv2450c | [2751484](http://genolist.pasteur.fr/TubercuList/genome.cgi?gene_list_from+from=2751.484+to=+location=kb+chkOrg0=2+TotOrg=1) | [2752420](http://genolist.pasteur.fr/TubercuList/genome.cgi?gene_list_from+from=2752.42+to=+location=kb+chkOrg0=2+TotOrg=1) | 936 | GCCCGGGCCAGGTATTC | CGGGCAGCAAGAAATCTCAACAAT |
| Rv2462c | [2763777](http://genolist.pasteur.fr/TubercuList/genome.cgi?gene_list_from+from=2763.777+to=+location=kb+chkOrg0=2+TotOrg=1) | [2765361](http://genolist.pasteur.fr/TubercuList/genome.cgi?gene_list_from+from=2765.361+to=+location=kb+chkOrg0=2+TotOrg=1) | 1584 | TGGGGCACCTGCTTTCCTC | AAGGCCGCAGGTTCAAATCC |
|  |  |  |  | 1) ATCGGCCAGGGCGTCTAGCAA |  |
| Rv2626c | [2952342](http://genolist.pasteur.fr/TubercuList/genome.cgi?gene_list_from+from=2952.342+to=+location=kb+chkOrg0=2+TotOrg=1) | [2953114](http://genolist.pasteur.fr/TubercuList/genome.cgi?gene_list_from+from=2953.114+to=+location=kb+chkOrg0=2+TotOrg=1) | 772 | ACCTGCGCCGAGAAGCCAATAGAC | CGCGCCAGTACGGGTCCATC |
| Rv2627c | [2953382](http://genolist.pasteur.fr/TubercuList/genome.cgi?gene_list_from+from=2953.382+to=+location=kb+chkOrg0=2+TotOrg=1) | [2954931](http://genolist.pasteur.fr/TubercuList/genome.cgi?gene_list_from+from=2954.931+to=+location=kb+chkOrg0=2+TotOrg=1) | 1549 | GCGGAGCCGGGTTGCCACATC | CCGCGACCAAGCCCCGTGAAAC |
|  |  |  |  | 1) CGGCCCAACAACTCGATCAGA |  |
| Rv2628 | [2954975](http://genolist.pasteur.fr/TubercuList/genome.cgi?gene_list_from+from=2954.975+to=+location=kb+chkOrg0=2+TotOrg=1) | [2955495](http://genolist.pasteur.fr/TubercuList/genome.cgi?gene_list_from+from=2955.495+to=+location=kb+chkOrg0=2+TotOrg=1) | 520 | GGCGCGACCGGGCACATC | GCGGGAAGGCATAGGGACCAAAGT |
| Rv2686c | [3003114](http://genolist.pasteur.fr/TubercuList/genome.cgi?gene_list_from+from=3003.114+to=+location=kb+chkOrg0=2+TotOrg=1) | [3004235](http://genolist.pasteur.fr/TubercuList/genome.cgi?gene_list_from+from=3004.235+to=+location=kb+chkOrg0=2+TotOrg=1) | 1121 | GCAGCCAGCGCCAATGTCGTCA | CTGTGGCCCAACCCGGTGCTTTAC |
| Rv2687c | [3003869](http://genolist.pasteur.fr/TubercuList/genome.cgi?gene_list_from+from=3003.869+to=+location=kb+chkOrg0=2+TotOrg=1) | [3004934](http://genolist.pasteur.fr/TubercuList/genome.cgi?gene_list_from+from=3004.934+to=+location=kb+chkOrg0=2+TotOrg=1) | 1065 | CGCGCCAGCATCTCGGTGAACAG | GCGGCGGGTGCGGGTGGAATAC |
| Rv2744c | [3057024](http://genolist.pasteur.fr/TubercuList/genome.cgi?gene_list_from+from=3057.024+to=+location=kb+chkOrg0=2+TotOrg=1) | [3058231](http://genolist.pasteur.fr/TubercuList/genome.cgi?gene_list_from+from=3058.231+to=+location=kb+chkOrg0=2+TotOrg=1) | 1207 | AGCGCCGTCACCAACCCCCAGAGC | GTCGTCATTGCGCCGGTGGTGTCG |
| Rv2873 | [3183659](http://genolist.pasteur.fr/TubercuList/genome.cgi?gene_list_from+from=3183.659+to=+location=kb+chkOrg0=2+TotOrg=1) | [3184755](http://genolist.pasteur.fr/TubercuList/genome.cgi?gene_list_from+from=3184.755+to=+location=kb+chkOrg0=2+TotOrg=1) | 1096 | GGCAGTTAGCGTCCGATGTTGATG | CAGCCCGGCGGTGTTGTTGC |
| Rv2875 | [3186865](http://genolist.pasteur.fr/TubercuList/genome.cgi?gene_list_from+from=3186.865+to=+location=kb+chkOrg0=2+TotOrg=1) | [3187857](http://genolist.pasteur.fr/TubercuList/genome.cgi?gene_list_from+from=3187.857+to=+location=kb+chkOrg0=2+TotOrg=1) | 992 | GCTGGCGTCCGAAACACTTGAGGT | CGCCAGCAGGAAGCCGAAGATG |
| Rv2924c | [3238442](http://genolist.pasteur.fr/TubercuList/genome.cgi?gene_list_from+from=3238.442+to=+location=kb+chkOrg0=2+TotOrg=1) | [3239845](http://genolist.pasteur.fr/TubercuList/genome.cgi?gene_list_from+from=3239.845+to=+location=kb+chkOrg0=2+TotOrg=1) | 1403 | GCGTGCTGCGTCCGATGTAAC | GCAAAACCTCCGCCTAACTGG |
|  |  |  |  | 1) GATGTTGCCGATTCCCGACAC |  |
| Rv2949c | [3299802](http://genolist.pasteur.fr/TubercuList/genome.cgi?gene_list_from+from=3299.802+to=+location=kb+chkOrg0=2+TotOrg=1) | [3300655](http://genolist.pasteur.fr/TubercuList/genome.cgi?gene_list_from+from=3300.655+to=+location=kb+chkOrg0=2+TotOrg=1) | 853 | TCCGCGGTTTCTCAGCACTTCC | TCGGCCAGCGTTGAGCGTTAC |
| Rv2952 | [3304242](http://genolist.pasteur.fr/TubercuList/genome.cgi?gene_list_from+from=3304.242+to=+location=kb+chkOrg0=2+TotOrg=1) | [3305423](http://genolist.pasteur.fr/TubercuList/genome.cgi?gene_list_from+from=3305.423+to=+location=kb+chkOrg0=2+TotOrg=1) | 1181 | CGCCCACGCCTACCCCTTGTCTAC | CGCCCCGCAGCCGTTCG |
| Rv2957 | [3309311](http://genolist.pasteur.fr/TubercuList/genome.cgi?gene_list_from+from=3309.311+to=+location=kb+chkOrg0=2+TotOrg=1) | [3310468](http://genolist.pasteur.fr/TubercuList/genome.cgi?gene_list_from+from=3310.468+to=+location=kb+chkOrg0=2+TotOrg=1) | 1157 | CGTTGACGGGATTGCTGCCTTGTT | CCGCCCACCCCAGATCCGAAATAG |
| Rv2958c | [3310491](http://genolist.pasteur.fr/TubercuList/genome.cgi?gene_list_from+from=3310.491+to=+location=kb+chkOrg0=2+TotOrg=1) | [3312131](http://genolist.pasteur.fr/TubercuList/genome.cgi?gene_list_from+from=3312.131+to=+location=kb+chkOrg0=2+TotOrg=1) | 1640 | GGTTTCGGGGGTTGCGGTGAGAC | CGCGCCCAACGGATTTCTGAT |
|  |  |  |  | 1) TGCCGCCACGCCTTCAGTGTT |  |
|  |  |  |  | 2) ACATCGCGGCTGACAGGAAGA |  |
| Rv2959c | [3311976](http://genolist.pasteur.fr/TubercuList/genome.cgi?gene_list_from+from=3311.976+to=+location=kb+chkOrg0=2+TotOrg=1) | [3312948](http://genolist.pasteur.fr/TubercuList/genome.cgi?gene_list_from+from=3312.948+to=+location=kb+chkOrg0=2+TotOrg=1) | 972 | CGCCGGCGACGCTTGTTTC | TTGCCGCAGCCATGTATTGAGTCC |
| Rv2962c | [3313620](http://genolist.pasteur.fr/TubercuList/genome.cgi?gene_list_from+from=3313.62+to=+location=kb+chkOrg0=2+TotOrg=1) | [3315365](http://genolist.pasteur.fr/TubercuList/genome.cgi?gene_list_from+from=3315.365+to=+location=kb+chkOrg0=2+TotOrg=1) | 1745 | GGCACGGCATCCTCACCCACAACC | CCGACAGCGCGAAGCCCAGGAT |
|  |  |  |  | 1) CTGCCACCCGCTGACTCTTCA |  |
|  |  |  |  | 2) TCGGGCACGTCAGCATACAGG |  |
| Rv2985 | [3342031](http://genolist.pasteur.fr/TubercuList/genome.cgi?gene_list_from+from=3342.031+to=+location=kb+chkOrg0=2+TotOrg=1) | [3343267](http://genolist.pasteur.fr/TubercuList/genome.cgi?gene_list_from+from=3343.267+to=+location=kb+chkOrg0=2+TotOrg=1) | 1236 | TAGCGTGCGCGACCATCAGGAATC | CGACCAAGGCGCCGGCAAAGAAG |
| Rv3126c | [3491560](http://genolist.pasteur.fr/TubercuList/genome.cgi?gene_list_from+from=3491.56+to=+location=kb+chkOrg0=2+TotOrg=1) | [3492330](http://genolist.pasteur.fr/TubercuList/genome.cgi?gene_list_from+from=3492.33+to=+location=kb+chkOrg0=2+TotOrg=1) | 770 | GAGGCCGCCGCAAACAAC | GTACGGCACCGCAACTGATGAT |
| Rv3129 | [3494494](http://genolist.pasteur.fr/TubercuList/genome.cgi?gene_list_from+from=3494.494+to=+location=kb+chkOrg0=2+TotOrg=1) | [3495234](http://genolist.pasteur.fr/TubercuList/genome.cgi?gene_list_from+from=3495.234+to=+location=kb+chkOrg0=2+TotOrg=1) | 740 | TGTTTCGCGGGCATTCTTGGTGAG | CACGACGGCCGCTGCAGATTATGG |
| Rv3132c | [3497416](http://genolist.pasteur.fr/TubercuList/genome.cgi?gene_list_from+from=3497.416+to=+location=kb+chkOrg0=2+TotOrg=1) | [3499437](http://genolist.pasteur.fr/TubercuList/genome.cgi?gene_list_from+from=3499.437+to=+location=kb+chkOrg0=2+TotOrg=1) | 2021 | CCGGCGGCTACCCCCAGATGC | TGCTTAGCGAGGGCCTGACCAACA |
|  |  |  |  | 1) GGAGCCGAGTGATACCCTGCG |  |
|  |  |  |  | 2) CAACAATGGAAGCCACAGCGC |  |
| Rv3133c | [3499180](http://genolist.pasteur.fr/TubercuList/genome.cgi?gene_list_from+from=3499.18+to=+location=kb+chkOrg0=2+TotOrg=1) | [3500165](http://genolist.pasteur.fr/TubercuList/genome.cgi?gene_list_from+from=3500.165+to=+location=kb+chkOrg0=2+TotOrg=1) | 985 | TCGTGCAGGCGTAGTTGGGAGAGC | GTTGGCGCATGTACACCTGAGC |
| Rv3221c | [3597396](http://genolist.pasteur.fr/TubercuList/genome.cgi?gene_list_from+from=3597.396+to=+location=kb+chkOrg0=2+TotOrg=1) | [3597869](http://genolist.pasteur.fr/TubercuList/genome.cgi?gene_list_from+from=3597.869+to=+location=kb+chkOrg0=2+TotOrg=1) | 473 | CGCGGCGAACCCACATCAGG | TGCCGCGCTCCCCAGGGTTCTTAT |
| Rv3261 | [3640449](http://genolist.pasteur.fr/TubercuList/genome.cgi?gene_list_from+from=3640.449+to=+location=kb+chkOrg0=2+TotOrg=1) | [3641714](http://genolist.pasteur.fr/TubercuList/genome.cgi?gene_list_from+from=3641.714+to=+location=kb+chkOrg0=2+TotOrg=1) | 1265 | TTTCCGCGGTTCTGTTGTGGTTGG | CAGCCGGCCCTCGCATTTGGACA |
| Rv3262 | [3641380](http://genolist.pasteur.fr/TubercuList/genome.cgi?gene_list_from+from=3641.38+to=+location=kb+chkOrg0=2+TotOrg=1) | [3643033](http://genolist.pasteur.fr/TubercuList/genome.cgi?gene_list_from+from=3643.033+to=+location=kb+chkOrg0=2+TotOrg=1) | 1653 | TGCGCCACCGGGATACTG | CGGCGCCTAAGTCCTGTCGT |
|  |  |  |  | 1) GGTCGATCCTGACGCCAGTGC |  |
| Rv3354 | [3768960](http://genolist.pasteur.fr/TubercuList/genome.cgi?gene_list_from+from=3768.96+to=+location=kb+chkOrg0=2+TotOrg=1) | [3769628](http://genolist.pasteur.fr/TubercuList/genome.cgi?gene_list_from+from=3769.628+to=+location=kb+chkOrg0=2+TotOrg=1) | 668 | CTCCGACGGCGCCGCGAAGAAAC | ACGGGCCGGCCTGTTGGTGGTG |
| Rv3407 | [3826012](http://genolist.pasteur.fr/TubercuList/genome.cgi?gene_list_from+from=3826.012+to=+location=kb+chkOrg0=2+TotOrg=1) | [3826736](http://genolist.pasteur.fr/TubercuList/genome.cgi?gene_list_from+from=3826.736+to=+location=kb+chkOrg0=2+TotOrg=1) | 724 | TGCCCGAAAACACCATCC | ACGCGCACGCTCAGTTTG |
| Rv3418c | [3836858](http://genolist.pasteur.fr/TubercuList/genome.cgi?gene_list_from+from=3836.858+to=+location=kb+chkOrg0=2+TotOrg=1) | [3837535](http://genolist.pasteur.fr/TubercuList/genome.cgi?gene_list_from+from=3837.535+to=+location=kb+chkOrg0=2+TotOrg=1) | 677 | GCGCGGTTTCGTCGTATTC | GTACCCGGACACGCTGGCAACC |
| Rv3547 | [3986648](http://genolist.pasteur.fr/TubercuList/genome.cgi?gene_list_from+from=3986.648+to=+location=kb+chkOrg0=2+TotOrg=1) | [3987493](http://genolist.pasteur.fr/TubercuList/genome.cgi?gene_list_from+from=3987.493+to=+location=kb+chkOrg0=2+TotOrg=1) | 845 | CGAGCGCACCGACCAGAGC | GCATGGCCCGCAGGTGGACAA |
| Rv3589 | [4030397](http://genolist.pasteur.fr/TubercuList/genome.cgi?gene_list_from+from=4030.397+to=+location=kb+chkOrg0=2+TotOrg=1) | [4031757](http://genolist.pasteur.fr/TubercuList/genome.cgi?gene_list_from+from=4031.757+to=+location=kb+chkOrg0=2+TotOrg=1) | 1360 | TCGACGCTCTGACTGGGATGCT | TTCGGCGACGGCGGTAAC |
|  |  |  |  | 1) GCCCGCGACCACAATGACGTG |  |
| Rv3763 | [4208964](http://genolist.pasteur.fr/TubercuList/genome.cgi?gene_list_from+from=4208.964+to=+location=kb+chkOrg0=2+TotOrg=1) | [4209693](http://genolist.pasteur.fr/TubercuList/genome.cgi?gene_list_from+from=4209.693+to=+location=kb+chkOrg0=2+TotOrg=1) | 729 | AGACCTCGGGCCCTGCTAACG | GGTCAAGGCGCACAACGGAACGAT |
| Rv3804c | [4265397](http://genolist.pasteur.fr/TubercuList/genome.cgi?gene_list_from+from=4265.397+to=+location=kb+chkOrg0=2+TotOrg=1) | [4266781](http://genolist.pasteur.fr/TubercuList/genome.cgi?gene_list_from+from=4266.781+to=+location=kb+chkOrg0=2+TotOrg=1) | 1384 | ACCGCCCAACCCGAATGACAGTG | TGGACGCCCGAAGTTGTGGTTGAC |
|  |  |  |  | 1) TCAGCGGCTGGGACATCAACA |  |
| Rv3872 | [4350591](http://genolist.pasteur.fr/TubercuList/genome.cgi?gene_list_from+from=4350.591+to=+location=kb+chkOrg0=2+TotOrg=1) | [4351124](http://genolist.pasteur.fr/TubercuList/genome.cgi?gene_list_from+from=4351.124+to=+location=kb+chkOrg0=2+TotOrg=1) | 533 | AGCGCCGGTTAAGATTATTTCATT | GCCATCAGCCGTGCGGTATTTAG |
| Rv3874/Rv3875 | [4352076](http://genolist.pasteur.fr/TubercuList/genome.cgi?gene_list_from+from=4352.076+to=+location=kb+chkOrg0=2+TotOrg=1) | [4353020](http://genolist.pasteur.fr/TubercuList/genome.cgi?gene_list_from+from=4353.02+to=+location=kb+chkOrg0=2+TotOrg=1) | 944 | GCAATCCGGCGGCTCCACCAG | TCGGCCGCCATGACAACCTCTC |
| Rv3890c/Rv3891c | [4373526](http://genolist.pasteur.fr/TubercuList/genome.cgi?gene_list_from+from=4373.526+to=+location=kb+chkOrg0=2+TotOrg=1) | [4374453](http://genolist.pasteur.fr/TubercuList/genome.cgi?gene_list_from+from=4374.453+to=+location=kb+chkOrg0=2+TotOrg=1) | 927 | GTCCAACCGCGGCAACAATG | GTCCGCAGAGCAGCCATACACTTC |
| Rv3904c/Rv3905c | [4390250](http://genolist.pasteur.fr/TubercuList/genome.cgi?gene_list_from+from=4390.25+to=+location=kb+chkOrg0=2+TotOrg=1) | [4391233](http://genolist.pasteur.fr/TubercuList/genome.cgi?gene_list_from+from=4391.233+to=+location=kb+chkOrg0=2+TotOrg=1) | 983 | GGCCGCCGAACCGTCATAGGAG | GGCTGGCCGATCGGGTGCTGTG |
| Rv3908 | [4393276](http://genolist.pasteur.fr/TubercuList/genome.cgi?gene_list_from+from=4393.276+to=+location=kb+chkOrg0=2+TotOrg=1) | [4394351](http://genolist.pasteur.fr/TubercuList/genome.cgi?gene_list_from+from=4394.351+to=+location=kb+chkOrg0=2+TotOrg=1) | 1075 | GCCCCCGACACAGCGACTTCT | GATCGATGCGGACCTGGACAAAAG |
