# Supplementary material for: High Functional Diversity in Mycobacterium tuberculosis Driven by Genetic Drift and Human Demography
Source: PLoS Biol. 2008 Dec 16;6(12):e311. doi: 10.1371/journal.pbio.0060311 (PMC2602723; doi:10.1371/journal.pbio.0060311)
Supplement: Supplementary file 7 [file pbio.0060311.st004.doc]

**Table S4.** SNPs identified in *M. canettii* strain K116.

| **Rv Nr.** | **SNP Name** | **Position** | **H37Rv Base Pair** | **Mutant Base Pair** | **Codon** | **H37Rv Amino Acid** | **Mutant Amino Acid** |
| --- | --- | --- | --- | --- | --- | --- | --- |
| 0005 | Rv0005_0330s | 0330 | gaG | gaA | 0110 | Glu | Glu |
| 0005 | Rv0005_1332s | 1332 | gtC | gtT | 0444 | Val | Val |
| 0005 | Rv0005_1971s | 1971 | gcT | gcC | 0657 | Ala | Ala |
| 0006 | Rv0006_0462s | 0462 | ccT | ccC | 0154 | Pro | Pro |
| 0006 | Rv0006_0534s | 0534 | tcA | tcG | 0178 | Ser | Ser |
| 0006 | Rv0006_0636s | 0636 | gaA | gaG | 0212 | Glu | Glu |
| 0006 | Rv0006_0669s | 0669 | gtT | gtC | 0223 | Val | Val |
| 0006 | Rv0006_0687s | 0687 | ccG | ccC | 0229 | Pro | Pro |
| 0006 | Rv0006_0738s | 0738 | acT | acC | 0246 | Thr | Thr |
| 0006 | Rv0006_0753s | 0753 | atT | atC | 0251 | Ile | Ile |
| 0006 | Rv0006_0897s | 0897 | atT | atC | 0299 | Ile | Ile |
| 0006 | Rv0006_0918s | 0918 | tcT | tcC | 0306 | Ser | Ser |
| 0006 | Rv0006_1098s | 1098 | gtT | gtC | 0366 | Val | Val |
| 0006 | Rv0006_1455s | 1455 | gaA | gaG | 0485 | Glu | Glu |
| 0006 | Rv0006_1509s | 1509 | ggA | ggC | 0503 | Gly | Gly |
| 0006 | Rv0006_1512n | 1512 | gaC | gaG | 0504 | Asp | Glu |
| 0006 | Rv0006_1665s | 1665 | gtC | gtG | 0555 | Val | Val |
| 0006 | Rv0006_1788s | 1788 | gtG | gtA | 0596 | Val | Val |
| 0006 | Rv0006_1800s | 1800 | ttA | ttG | 0600 | Leu | Leu |
| 0006 | Rv0006_1881s | 1881 | acT | acC | 0627 | Thr | Thr |
| 0006 | Rv0006_1899s | 1899 | aaA | aaG | 0633 | Lys | Lys |
| 0006 | Rv0006_2076s | 2076 | ccA | ccG | 0692 | Pro | Pro |
| 0006 | Rv0006_2415s | 2415 | aaT | aaC | 0805 | Asn | Asn |
| 0006 | Rv0006_2439s | 2439 | ttG | ttA | 0813 | Leu | Leu |
| 0006 | Rv0006_2475s | 2475 | gaT | gaC | 0825 | Asp | Asp |
| 0006 | Rv0006_2517s | 2517 | taA | taG | 0839 | TER | TER |
| 0009 | Rv0009_0045s | 0045 | gcT | gcC | 0015 | Ala | Ala |
| 0009 | Rv0009_0354s | 0354 | gcC | gcT | 0118 | Ala | Ala |
| 0009 | Rv0009_0537s | 0537 | atC | atT | 0179 | Ile | Ile |
| 0040c | Rv0040c_0258s | 0258 | ccC | ccG | 0086 | Pro | Pro |
| 0040c | Rv0040c_0358n | 0358 | Cag | Gag | 0120 | Gln | Glu |
| 0040c | Rv0040c_0513s | 0510 | taT | taC | 0171 | Tyr | Tyr |
| 0040c | Rv0040c_0515n | 0515 | aGg | aAg | 0172 | Arg | Lys |
| 0040c | Rv0040c_0549s | 0549 | atC | atT | 0183 | Ile | Ile |
| 0079 | Rv0079_0389n | 0389 | cCg | cTg | 0130 | Pro | Leu |
| 0164 | Rv0164_0014n | 0014 | tCg | tTg | 0005 | Ser | Leu |
| 0251c | Rv0251c_0437n | 0437 | gCc | gAc | 0146 | Ala | Asp |
| 0251c | Rv0251c_0468s | 0468 | gcC | gcT | 0156 | Ala | Ala |
| 0287 | Rv0287_0165s | 0165 | caT | caC | 0055 | His | His |
| 0407 | Rv0407_0399s | 0399 | cgT | cgC | 0133 | Arg | Arg |
| 0407 | Rv0407_0409n | 0409 | Ggg | Cgg | 0137 | Gly | Arg |
| 0407 | Rv0407_0414s | 0414 | ctA | ctG | 0138 | Leu | Leu |
| 0407 | Rv0407_0430n | 0430 | Agc | Cgc | 0144 | Ser | Arg |
| 0429c | Rv0429c_0486s | 0486 | ctC | ctT | 0162 | Leu | Leu |
| 0475 | Rv0475_0525s | 0525 | gcC | gcT | 0175 | Ala | Ala |
| 0934 | Rv0934_0414s | 0414 | ggA | ggT | 0138 | Gly | Gly |
| 1009 | Rv1009_0825n | 0825 | gaG | gaC | 0275 | Glu | Asp |
| 1009 | Rv1009_1034n | 1034 | cTg | cAg | 0345 | Leu | Gln |
| 1009 | Rv1009_1038s | 1038 | cgT | cgC | 0346 | Arg | Arg |
| 1160 | Rv1160_0005n | 0005 | cTg | cCg | 0002 | Leu | Pro |
| 1160 | Rv1160_0093s | 0093 | cgT | cgC | 0031 | Arg | Arg |
| 1173 | Rv1173_0765s | 0765 | ttT | ttC | 0255 | Phe | Phe |
| 1173 | Rv1173_1668s | 1668 | aaC | aaT | 0556 | Asn | Asn |
| 1195 | Rv1195_0237s | 0237 | acC | acG | 0079 | Thr | Thr |
| 1411c | Rv1411c_0679n | 0679 | Aag | Gag | 0227 | Lys | Glu |
| 1733c | Rv1733c_0070n | 0070 | Acg | Ccg | 0024 | Thr | Pro |
| 1733c | Rv1733c_0298n | 0298 | Acg | Gcg | 0100 | Thr | Ala |
| 1733c | Rv1733c_0484n | 0484 | Gcg | Acg | 0162 | Ala | Thr |
| 1733c | Rv1733c_0486s | 0486 | gcG | gcC | 0162 | Ala | Ala |
| 1884c | Rv1884c_0004n | 0004 | Cat | Tat | 0002 | His | Tyr |
| 1980c | Rv1980c_0134n | 0134 | aTg | aCg | 0045 | Met | Thr |
| 1996 | Rv1996_0450s | 0450 | caC | caT | 0150 | His | His |
| 2030c | Rv2030c_1175n | 1175 | aAg | aCg | 0392 | Lys | Thr |
| 2032 | Rv2032_0186s | 0186 | ggG | ggC | 0032 | Gly | Gly |
| 2032 | Rv2032_0813s | 0813 | acG | acC | 0271 | Thr | Thr |
| 2185c | Rv2185c_0378s | 0378 | gcG | gcT | 0126 | Ala | Ala |
| 2450c | Rv2450c_0315s | 0315 | gcG | gcA | 0105 | Ala | Ala |
| 2450c | Rv2450c_0417s | 0417 | ggG | ggA | 0139 | Gly | Gly |
| 2627c | Rv2627c_0029n | 0029 | cAc | cGc | 0011 | His | Arg |
| 2627c | Rv2627c_0137n | 0137 | cAa | cGa | 0046 | Gln | Arg |
| 2627c | Rv2627c_0429s | 0429 | ttT | ttC | 0143 | Phe | Phe |
| 2627c | Rv2627c_0934n | 0934 | Aaa | Gaa | 0312 | Lys | Glu |
| 2628 | Rv2628_0078s | 0078 | atA | atC | 0026 | Ile | Ile |
| 2628 | Rv2628_0145n | 0145 | Tcc | Gcc | 0049 | Ser | Ala |
| 2628 | Rv2628_0253n | 0253 | Acc | Gcc | 0085 | Thr | Ala |
| 2628 | Rv2628_0273s | 0273 | gaT | gaC | 0091 | Asp | Asp |
| 2686c | Rv2686c_0066s | 0066 | cgA | cgC | 0022 | Arg | Arg |
| 2686c | Rv2686c_0099s | 0099 | ctC | ctG | 0033 | Leu | Leu |
| 2686c | Rv2686c_0103n | 0103 | Atc | Gtc | 0035 | Ile | Val |
| 2686c | Rv2686c_0108s | 0108 | gcA | gcG | 0036 | Ala | Ala |
| 2686c | Rv2686c_0132s | 0132 | gtC | gtG | 0044 | Val | Val |
| 2686c | Rv2686c_0136n | 0136 | Ctg | Gtg | 0046 | Leu | Val |
| 2686c | Rv2686c_0141s | 0141 | ctC | ctA | 0047 | Leu | Leu |
| 2686c | Rv2686c_0148s | 0148 | Ctg | Ttg | 0050 | Leu | Leu |
| 2686c | Rv2686c_0282n | 0282 | gaC | gaG | 0094 | Asp | Glu |
| 2686c | Rv2686c_0285s | 0285 | gtA | gtG | 0095 | Val | Val |
| 2686c | Rv2686c_0301n | 0301 | Acg | Gcg | 0101 | Thr | Ala |
| 2686c | Rv2686c_0312s | 0312 | cgA | cgG | 0104 | Arg | Arg |
| 2686c | Rv2686c_0315s | 0315 | gtA | gtC | 0105 | Val | Val |
| 2686c | Rv2686c_0482n | 0482 | aCg | aTg | 0161 | Thr | Met |
| 2686c | Rv2686c_0544s | 0544 | Ctg | Ttg | 0182 | Leu | Leu |
| 2686c | Rv2686c_0565s | 0565 | Ctg | Ttg | 0189 | Leu | Leu |
| 2686c | Rv2686c_0608n | 0608 | gCg | gTg | 0203 | Ala | Val |
| 2687c | Rv2687c_0162s | 0162 | gaT | gaC | 0054 | Asp | Asp |
| 2687c | Rv2687c_0169n | 0169 | Atc | Gtc | 0057 | Ile | Val |
| 2687c | Rv2687c_0417s | 0417 | agT | agC | 0139 | Ser | Ser |
| 2687c | Rv2687c_0537s | 0537 | taC | taT | 0179 | Tyr | Tyr |
| 2687c | Rv2687c_0546s | 0546 | ccC | ccT | 0182 | Pro | Pro |
| 2873 | Rv2873_0026n | 0026 | gCc | gTc | 0009 | Ala | Val |
| 2875 | Rv2875_0360s | 0360 | aaT | aaC | 0120 | Asn | Asn |
| 2949c | Rv2949c_0093s | 0093 | gtT | gtG | 0031 | Val | Val |
| 2952 | Rv2952_0557n | 0557 | aAc | aGc | 0186 | Asn | Ser |
| 2952 | Rv2952_0723s | 0723 | cgC | cgT | 0241 | Arg | Arg |
| 2959c | Rv2959c_0621s | 0621 | ccC | ccT | 0207 | Pro | Pro |
| 2962c | Rv2962c_0978s | 0978 | gtG | gtC | 0326 | Val | Val |
| 3129 | Rv3129_0004n | 0004 | Gtg | Atg | 0002 | Val | Met |
| 3129 | Rv3129_0164n | 0164 | gGg | gAg | 0055 | Gly | Glu |
| 3132c | Rv3132c_1244n | 1244 | cAc | cGc | 0415 | His | Arg |
| 3132c | Rv3132c_1296s | 1296 | ctG | ctT | 0432 | Leu | Leu |
| 3354 | Rv3354_0065n | 0065 | aGc | gCc | 0022 | Ser | Thr |
| 3407 | Rv3407_0105s | 0105 | gtC | gtT | 0035 | Val | Val |
| 3418c | Rv3418c_0192s | 0192 | gcG | gcC | 0064 | Ala | Ala |
| 3547 | Rv3547_0067s | 0067 | Cgg | Agg | 0023 | Arg | Arg |
| 3547 | Rv3547_0207s | 0207 | gaC | gaT | 0069 | Asp | Asp |
| 3547 | Rv3547_0444s | 0444 | gtT | gtC | 0148 | Val | Val |
| 3589 | Rv3589_0715n | 0715 | Acc | Tcc | 0239 | Thr | Ser |
| 3763 | Rv3763_0267s | 0267 | ctC | ctT | 0089 | Leu | Leu |
| 3872 | Rv3872_0147n | 0147 | caA | caT | 0048 | Gln | His |
