# Supplementary material for: High Functional Diversity in Mycobacterium tuberculosis Driven by Genetic Drift and Human Demography
Source: PLoS Biol. 2008 Dec 16;6(12):e311. doi: 10.1371/journal.pbio.0060311 (PMC2602723; doi:10.1371/journal.pbio.0060311)
Supplement: Supplementary file 8 [file pbio.0060311.st005.doc]

**Table S5.** SNPs identified in 107 *M. tuberculosis* complex strains.

| **Rv Nr.** | **SNP Name** | **Position** | **H37Rv Base Pair** | **Mutant Base Pair** | **Codon** | **H37Rv Amino Acid** | **Mutant Amino Acid** |
| --- | --- | --- | --- | --- | --- | --- | --- |
| 0005 | Rv0005_0398n | 0398 | cCg | cTg | 0133 | Pro | Leu |
| 0005 | Rv0005_0549s | 0549 | taC | taT | 0183 | Tyr | Tyr |
| 0005 | Rv0005_0630s | 0630 | gtG | gtA | 0210 | Val | Val |
| 0005 | Rv0005_0990n | 0990 | atG | atC | 0330 | Met | Ile |
| 0005 | Rv0005_1002s | 1002 | gcC | gcT | 0334 | Ala | Ala |
| 0005 | Rv0005_1128s | 1128 | ctA | ctG | 0376 | Leu | Leu |
| 0005 | Rv0005_1185s | 1185 | gcT | gcG | 0395 | Ala | Ala |
| 0005 | Rv0005_1284s | 1284 | aaC | aaT | 0428 | Asn | Asn |
| 0005 | Rv0005_1316n | 1316 | cCc | cGc | 0439 | Pro | Arg |
| 0005 | Rv0005_1324n | 1324 | Gcg | Tcg | 0442 | Ala | Ser |
| 0005 | Rv0005_1599s | 1599 | gaC | gaT | 0533 | Asp | Asp |
| 0005 | Rv0005_1676n | 1676 | gGc | gCc | 0559 | Gly | Ala |
| 0005 | Rv0005_1695s | 1695 | aaG | aaA | 0565 | Lys | Lys |
| 0005 | Rv0005_2099n | 0700 | aGc | aCc | 0700 | Ser | Thr |
| 0005 | Rv0005_2100s | 2100 | agC | agT | 0700 | Ser | Ser |
| 0006 | Rv0006_0061n | 0061 | Gag | Cag | 0021 | Glu | Gln |
| 0006 | Rv0006_0238n | 0238 | Acc | Gcc | 0080 | Thr | Ala |
| 0006 | Rv0006_0284n | 0284 | aGc | aCc | 0095 | Ser | Thr |
| 0006 | Rv0006_0591s | 0591 | ctG | ctA | 0197 | Leu | Leu |
| 0006 | Rv0006_0670n | 0670 | Aaa | Gaa | 0224 | Lys | Glu |
| 0006 | Rv0006_0739n | 0739 | Ggc | Agc | 0247 | Gly | Ser |
| 0006 | Rv0006_0774n | 0774 | gaG | gaT | 0258 | Glu | Asp |
| 0006 | Rv0006_0887n | 0887 | cTg | cCg | 0296 | Leu | Pro |
| 0006 | Rv0006_0984s | 0984 | atC | atT | 0318 | Ile | Ile |
| 0006 | Rv0006_1036n | 1036 | Cta | Gta | 0346 | Leu | Val |
| 0006 | Rv0006_1151n | 1151 | gCa | gTa | 0384 | Ala | Val |
| 0006 | Rv0006_1192n | 1192 | Ctg | Ttc | 0398 | Leu | Phe |
| 0006 | Rv0006_1207n | 1207 | Gag | Cag | 0403 | Glu | Gln |
| 0006 | Rv0006_1314n | 1314 | atG | atC | 0438 | Met | Ile |
| 0006 | Rv0006_1389s | 1389 | gcC | gcG | 0463 | Ala | Ala |
| 0006 | Rv0006_1440s | 1440 | cgC | cgT | 0480 | Arg | Arg |
| 0006 | Rv0006_1505n | 1505 | gAc | gGc | 0502 | Asp | Gly |
| 0006 | Rv0006_1647s | 1647 | ttG | ttA | 0549 | Leu | Leu |
| 0006 | Rv0006_1746s | 1746 | taC | taT | 0582 | Tyr | Tyr |
| 0006 | Rv0006_1842s | 1842 | atT | atC | 0614 | Ile | Ile |
| 0006 | Rv0006_1916n | 1916 | gAc | gCc | 0639 | Asp | Ala |
| 0006 | Rv0006_1959s | 1959 | ctG | ctC | 0653 | Leu | Leu |
| 0006 | Rv0006_2003n | 2003 | gGc | gAc | 0668 | Gly | Asp |
| 0006 | Rv0006_2142s | 2142 | ctG | ctA | 0714 | Leu | Leu |
| 0006 | Rv0006_2265s | 2265 | taC | taT | 0755 | Tyr | Tyr |
| 0006 | Rv0006_2295s | 2295 | gcG | gcT | 0765 | Ala | Ala |
| 0006 | Rv0006_2328s | 2328 | gcC | gcT | 0776 | Ala | Ala |
| 0006 | Rv0006_2428n | 2428 | Gac | Cac | 0810 | Asp | His |
| 0009 | Rv0009_0054s | 0054 | acG | acA | 0018 | Thr | Thr |
| 0009 | Rv0009_0088s | 0088 | Ctg | Ttg | 0030 | Leu | Leu |
| 0009 | Rv0009_0387s | 0387 | atC | atT | 0129 | Ile | Ile |
| 0009 | Rv0009_0492s | 0492 | acC | acG | 0164 | Thr | Thr |
| 0009 | Rv0009_0496n | 0496 | Ggc | Agc | 0166 | Gly | Ser |
| 0040c | Rv0040c_0175n | 0175 | Gcg | Tcg | 0059 | Ala | Ser |
| 0040c | Rv0040c_0399s | 0399 | ccC | ccG | 0133 | Pro | Pro |
| 0040c | Rv0040c_0506n | 0506 | gTg | gCg | 0169 | Val | Ala |
| 0040c | Rv0040c_0555s | 0555 | caC | caT | 0185 | His | His |
| 0040c | Rv0040c_0610n | 0610 | Gcc | Acc | 0204 | Ala | Thr |
| 0040c | Rv0040c_0619n | 0619 | Gac | Aac | 0207 | Asp | Asn |
| 0040c | Rv0040c_0876s | 0876 | ccC | ccT | 0876 | Pro | Pro |
| 0079 | Rv0079_0049n | 0049 | Cac | Aac | 0017 | His | Asn |
| 0079 | Rv0079_0231s | 0231 | ctG | ctT | 0077 | Leu | Leu |
| 0079 | Rv0079_0256n | 0256 | Gcg | Acg | 0086 | Ala | Thr |
| 0079 | Rv0079_0570n | 0570 | caC | caG | 0190 | His | Gln |
| 0079 | Rv0079_0661n | 0661 | Acg | Gcg | 0221 | Thr | Ala |
| 0129c | Rv0129c_0309s | 0309 | gaG | gaA | 0103 | Glu | Glu |
| 0129c | Rv0129c_0472n | 0472 | Ggc | Agc | 0158 | Gly | Ser |
| 0129c | Rv0129c_0869n | 0869 | gCc | gTc | 0290 | Ala | Val |
| 0164 | Rv0164_0405n | 0405 | atG | atA | 0135 | Met | Ile |
| 0164 | Rv0164_0415n | 0415 | Ctg | Atg | 0139 | Leu | Met |
| 0164 | Rv0164_0432s | 0432 | ctG | ctT | 0144 | Leu | Leu |
| 0251c | Rv0251c_0038n | 0038 | gAc | gGc | 0013 | Asp | Gly |
| 0251c | Rv0251c_0124n | 0124 | Acg | Gcg | 0042 | Thr | Ala |
| 0251c | Rv0251c_0349n | 0349 | Cgg | Tgg | 0117 | Arg | Trp |
| 0251c | Rv0251c_0395n | 0395 | gAc | gCc | 0132 | Asp | Ala |
| 0287 | Rv0287_0106n | 0106 | Gcg | Ccg | 0036 | Ala | Pro |
| 0287 | Rv0287_0166n | 0166 | Gcc | Acc | 0056 | Ala | Thr |
| 0288 | Rv0288_0026n | 0026 | cCc | cTc | 0009 | Pro | Leu |
| 0288 | Rv0288_0028n | 0028 | Gcg | Acg | 0010 | Ala | Thr |
| 0288 | Rv0288_0038n | 0038 | gGt | gAt | 0013 | Gly | Asp |
| 0288 | Rv0288_0080n | 0080 | aGc | aAc | 0027 | Ser | Asn |
| 0288 | Rv0288_0211n | 0211 | Gcg | Tcg | 0071 | Ala | Ser |
| 0288 | Rv0288_0220n | 0220 | Agc | Cgc | 0074 | Ser | Arg |
| 0407 | Rv0407_0190n | 0190 | Cgt | Agt | 0064 | Arg | Ser |
| 0407 | Rv0407_0660s | 0660 | cgA | cgG | 0220 | Arg | Arg |
| 0407 | Rv0407_0809n | 0809 | aAg | aTg | 0270 | Lys | Met |
| 0407 | Rv0407_0886n | 0886 | Aag | Ggg | 0296 | Lys | Glu |
| 0407 | Rv0407_0960s | 0960 | ttT | ttC | 0320 | Phe | Phe |
| 0410c | Rv0410c_0174n | 0174 | caA | caC | 0058 | Gln | His |
| 0410c | Rv0410c_0207s | 0207 | ccG | ccA | 0069 | Pro | Pro |
| 0410c | Rv0410c_0315n | 0315 | ttC | ttA | 0105 | Phe | Leu |
| 0410c | Rv0410c_0389n | 0389 | tAt | tCt | 0130 | Tyr | Ser |
| 0410c | Rv0410c_0433n | 0433 | Gac | Cac | 0145 | Asp | His |
| 0410c | Rv0410c_0475n | 0475 | Cac | Tac | 0159 | His | Tyr |
| 0410c | Rv0410c_1428s | 1428 | ccG | ccC | 0476 | Pro | Pro |
| 0410c | Rv0410c_1842s | 1842 | tcG | tcA | 0614 | Ser | Ser |
| 0410c | Rv0410c_1993n | 1993 | Gcc | Acc | 0665 | Ala | Thr |
| 0410c | Rv0410c_2117n | 2117 | tTc | tCc | 0706 | Phe | Ser |
| 0410c | Rv0410c_2207n | 2207 | aCg | aAg | 0736 | Thr | Lys |
| 0410c | Rv0410c_2247s | 2247 | acG | acA | 0749 | Thr | Thr |
| 0413 | Rv0413_0198s | 0198 | ggC | ggT | 0066 | Gly | Gly |
| 0413 | Rv0413_0433n | 0433 | Gag | Aag | 0145 | Glu | Lys |
| 0413 | Rv0413_0511n | 0511 | Gtg | Ttg | 0171 | Val | Leu |
| 0413 | Rv0413_0523n | 0523 | Cgg | Tgg | 0175 | Arg | Trp |
| 0413 | Rv0413_0627s | 0627 | ctG | ctA | 0209 | Leu | Leu |
| 0429c | Rv0429c_0211n | 0211 | Gac | Aac | 0071 | Asp | Asn |
| 0429c | Rv0429c_0231s | 0231 | cgC | cgA | 0077 | Arg | Arg |
| 0467 | Rv0467_0015s | 0015 | ggC | ggT | 0005 | Gly | Gly |
| 0467 | Rv0467_0549s | 0549 | gaC | gaT | 0183 | Asp | Asp |
| 0467 | Rv0467_0799n | 0799 | Cct | Gct | 0267 | Pro | Ala |
| 0467 | Rv0467_0814n | 0814 | Gcg | Acg | 0272 | Ala | Thr |
| 0467 | Rv0467_0975s | 0975 | gaC | gaT | 0325 | Asp | Asp |
| 0467 | Rv0467_1057n | 1057 | atG | atA | 0353 | Met | Ile |
| 0467 | Rv0467_1194s | 1194 | ggC | ggT | 0398 | Gly | Gly |
| 0467 | Rv0467_1224s | 1224 | acC | acT | 0408 | Thr | Thr |
| 0475 | Rv0475_0013n | 0013 | Tcg | Acg | 0005 | Ser | Thr |
| 0475 | Rv0475_0393s | 0393 | gaG | gaA | 0131 | Glu | Glu |
| 0475 | Rv0475_0468s | 0468 | ggC | ggG | 0156 | Gly | Gly |
| 0475 | Rv0475_0532n | 0532 | Gcc | Acc | 0178 | Ala | Thr |
| 0569 | Rv0569_0124n | 0124 | Ctc | Ttc | 0042 | Leu | Phe |
| 0652 | Rv0652_0065n | 0065 | tCc | tTc | 0022 | Ser | Phe |
| 0652 | Rv0652_0328n | 0328 | Aag | Gag | 0110 | Lys | Glu |
| 0798c | Rv0798c_0200n | 0200 | gCc | gTc | 0067 | Ala | Val |
| 0798c | Rv0798c_0514s | 0514 | Ttg | Ctg | 0172 | Leu | Leu |
| 0798c | Rv0798c_0516s | 0516 | ttG | ttA | 0172 | Leu | Leu |
| 0909 | Rv0909_0135n | 0135 | caT | caG | 0045 | His | Gln |
| 0934 | Rv0934_0900n | 0900 | atG | atC | 0300 | Met | Ile |
| 0934 | Rv0934_1022n | 1022 | aCc | aTc | 0341 | Thr | Ile |
| 0934 | Rv0934_1055n | 1055 | gTt | gCt | 0352 | Val | Ala |
| 1009 | Rv1009_0055n | 0055 | Tat | Gat | 0019 | Tyr | Asp |
| 1009 | Rv1009_0136n | 0136 | Atc | Gtc | 0046 | Ile | Val |
| 1009 | Rv1009_0714s | 0714 | ttC | ttT | 0238 | Phe | Phe |
| 1009 | Rv1009_0724n | 0724 | Gag | Aag | 0242 | Glu | Lys |
| 1009 | Rv1009_0735s | 0735 | ggC | ggT | 0245 | Gly | Gly |
| 1009 | Rv1009_0793n | 0793 | Gtg | Atg | 0265 | Val | Met |
| 1009 | Rv1009_0841n | 0841 | Gac | Aac | 0281 | Asp | Asn |
| 1009 | Rv1009_0845n | 0845 | gGa | gAa | 0282 | Gly | Glu |
| 1009 | Rv1009_1070n | 1070 | gCa | gTa | 0357 | Ala | Val |
| 1072 | Rv1072_0230n | 0230 | gCg | gTg | 0077 | Ala | Val |
| 1072 | Rv1072_0493n | 0493 | Aca | Gca | 0165 | Thr | Ala |
| 1160 | Rv1160_0172n | 0172 | Gga | Cga | 0058 | Gly | Arg |
| 1160 | Rv1160_0233n | 0233 | gGc | gCc | 0078 | Gly | Ala |
| 1173 | Rv1173_0235n | 0235 | Agc | Cgc | 0079 | Ser | Arg |
| 1173 | Rv1173_1513n | 1513 | Gcg | Acg | 0505 | Ala | Thr |
| 1173 | Rv1173_2263n | 2263 | Ggt | Agt | 0755 | Gly | Ser |
| 1174c | Rv1174c_0299n | 0299 | gTc | gCc | 0100 | Val | Ala |
| 1184c | Rv1184c_0765s | 0765 | ggA | ggG | 0255 | Gly | Gly |
| 1184c | Rv1184c_0888n | 0888 | atG | atT | 0296 | Met | Ile |
| 1184c | Rv1184c_1047s | 1047 | cgC | cgT | 0349 | Arg | Arg |
| 1305 | Rv1305_0103n | 0103 | Ctt | Ttt | 0035 | Leu | Phe |
| 1305 | Rv1305_0183s | 0183 | gaG | gaA | 0061 | Glu | Glu |
| 1305 | Rv1305_0207s | 0207 | gcG | gcT | 069 | Ala | Ala |
| 1316c | Rv1316c_0036s | 0036 | ggG | ggA | 0012 | Gly | Gly |
| 1316c | Rv1316c_0044n | 0044 | aCc | aGc | 0015 | Thr | Ser |
| 1316c | Rv1316c_0134n | 0134 | gGc | gCc | 0045 | Gly | Ala |
| 1411c | Rv1411c_0027s | 0027 | cgT | cgC | 0009 | Arg | Arg |
| 1411c | Rv1411c_0510s | 0510 | ggG | ggA | 0170 | Gly | Gly |
| 1411c | Rv1411c_0658n | 0658 | Acc | Gcc | 0220 | Thr | Ala |
| 1636 | Rv1636_0361n | 0361 | Gcg | Tcg | 0121 | Ala | Ser |
| 1733c | Rv1733c_0003n | 0003 | atG | atA | 0001 | Met | Ile |
| 1733c | Rv1733c_0097n | 0097 | Ccg | Tcg | 0033 | Pro | Ser |
| 1733c | Rv1733c_0204n | 0204 | caG | caT | 0068 | Gln | His |
| 1733c | Rv1733c_0303s | 0303 | acC | acT | 0101 | Thr | Thr |
| 1733c | Rv1733c_0471s | 0471 | ccG | ccA | 0157 | Pro | Pro |
| 1813c | Rv1813c_0144s | 0144 | gcC | gcA | 0048 | Ala | Ala |
| 1813c | Rv1813c_0304s | 0304 | Agg | Cgg | 0102 | Arg | Arg |
| 1813c | Rv1813c_0367n | 0367 | Gcg | Acg | 0123 | Ala | Thr |
| 1884c | Rv1884c_0047n | 0047 | cAc | cGc | 0016 | His | Arg |
| 1886c | Rv1886c_0052n | 0052 | Ggc | Cgc | 0018 | Gly | Arg |
| 1886c | Rv1886c_0132s | 0132 | ccG | ccC | 0044 | Pro | Pro |
| 1886c | Rv1886c_0344n | 0344 | cAg | cCg | 0115 | Gln | Pro |
| 1886c | Rv1886c_0399n | 0399 | caG | caC | 0133 | Gln | His |
| 1886c | Rv1886c_0584n | 0584 | cCc | cTc | 0195 | Pro | Leu |
| 1886c | Rv1886c_0714s | 0714 | ccC | ccA | 0238 | Pro | Pro |
| 1926c | Rv1926c_0095n | 0095 | cCc | cTc | 0032 | Pro | Leu |
| 1926c | Rv1926c_0263n | 0263 | aGc | aAc | 0088 | Ser | Asn |
| 1980c | Rv1980c_0128n | 0128 | aTt | aAt | 0043 | Ile | Asn |
| 1980c | Rv1980c_0348s | 0348 | gtC | gtG | 0116 | Val | Val |
| 1980c | Rv1980c_0555s | 0555 | gtG | gtT | 0185 | Val | Val |
| 1984c | Rv1984c_0523n | 0523 | Aac | Gac | 0175 | Asn | Asp |
| 1996 | Rv1996_0052n | 0052 | Ccc | Tcc | 0018 | Pro | Ser |
| 1996 | Rv1996_0153s | 0153 | ccG | ccT | 0051 | Pro | Pro |
| 1996 | Rv1996_0157n | 0157 | Ggg | Cgg | 0053 | Gly | Arg |
| 1996 | Rv1996_0346n | 0346 | Gcc | Acc | 0116 | Ala | Thr |
| 1996 | Rv1996_0381s | 0381 | ctG | ctA | 0127 | Leu | Leu |
| 2007c | Rv2007c_0080n | 0080 | tAt | tGt | 0027 | Tyr | Cys |
| 2030c | Rv2030c_0045s | 0045 | cgG | cgC | 0015 | Arg | Arg |
| 2030c | Rv2030c_0061n | 0061 | Cgc | Agc | 0021 | Arg | Ser |
| 2030c | Rv2030c_0111s | 0111 | gaC | gaT | 0037 | Asp | Asp |
| 2030c | Rv2030c_0824n | 0824 | aGc | aAc | 0275 | Ser | Asn |
| 2030c | Rv2030c_1137s | 1137 | ctG | ctA | 0379 | Leu | Leu |
| 2030c | Rv2030c_1569s | 1569 | caT | caC | 0523 | His | His |
| 2030c | Rv2030c_1670n | 1670 | aGc | aAc | 0557 | Ser | Asn |
| 2030c | Rv2030c_1753n | 1753 | Gca | Tca | 0585 | Ala | Ser |
| 2031c | Rv2031c_0018s | 0018 | ccC | ccT | 0006 | Pro | Pro |
| 2031c | Rv2031c_0426s | 0426 | tcC | tcT | 0142 | Ser | Ser |
| 2032 | Rv2032_0100n | 0100 | Gcc | Tcc | 0034 | Ala | Ser |
| 2032 | Rv2032_0381s | 0381 | ttC | ttT | 0127 | Phe | Phe |
| 2032 | Rv2032_0536n | 0536 | tAt | tGt | 0179 | Tyr | Cys |
| 2032 | Rv2032_0555n | 0555 | tgG | tgT | 0185 | Trp | Cys |
| 2032 | Rv2032_0630s | 0630 | gtC | gtT | 0210 | Val | Val |
| 2032 | Rv2032_0632n | 0632 | aCc | aTc | 0211 | Thr | Ile |
| 2032 | Rv2032_0759s | 0759 | gaG | gaA | 0253 | Glu | Glu |
| 2032 | Rv2032_0953n | 0953 | cCa | cTa | 0318 | Pro | Leu |
| 2185c | Rv2185c_0075s | 0075 | atC | atA | 0025 | Ile | Ile |
| 2185c | Rv2185c_0146n | 0146 | cCg | cTg | 0049 | Pro | Leu |
| 2185c | Rv2185c_0321s | 0321 | acC | acA | 0107 | Thr | Thr |
| 2185c | Rv2185c_0351s | 0351 | ccC | ccA | 0117 | Pro | Pro |
| 2330c | Rv2330c_0199n | 0199 | Gtg | Atg | 0067 | Val | Met |
| 2330c | Rv2330c_0287n | 0287 | gCc | gTc | 0096 | Ala | Val |
| 2330c | Rv2330c_0426s | 0426 | atC | atT | 0142 | Ile | Ile |
| 2330c | Rv2330c_0487n | 0487 | Gat | Aat | 0163 | Asp | Asn |
| 2450c | Rv2450c_0049s | 0049 | Ttg | Ctg | 0017 | Leu | Leu |
| 2450c | Rv2450c_0059n | 0059 | aCg | aGg | 0020 | Thr | Arg |
| 2450c | Rv2450c_0086n | 0086 | gAc | gGc | 0029 | Asp | Gly |
| 2450c | Rv2450c_0285s | 0285 | gtG | gtA | 0095 | Val | Val |
| 2450c | Rv2450c_0343n | 0343 | Atc | Gtc | 0115 | Ile | Val |
| 2450c | Rv2450c_0377n | 0377 | cGg | cAg | 0126 | Arg | Gln |
| 2462c | Rv2462c_0353n | 0353 | cCc | cTc | 0118 | Pro | Leu |
| 2462c | Rv2462c_0548n | 0548 | gCt | gGt | 0183 | Ala | Gly |
| 2462c | Rv2462c_0855s | 855 | gcC | gcT | 0285 | Ala | Ala |
| 2462c | Rv2462c_0934n | 0934 | Cac | Gac | 0312 | His | Asp |
| 2462c | Rv2462c_0979n | 0979 | Gag | Aag | 0327 | Glu | Lys |
| 2462c | Rv2462c_1086s | 1086 | gaT | gaC | 0362 | Asp | Asp |
| 2462c | Rv2462c_1119s | 1119 | ctG | ctA | 0373 | Leu | Leu |
| 2626c | Rv2626c_0072s | 0072 | gcC | gcA | 0024 | Ala | Ala |
| 2626c | Rv2626c_0093s | 0093 | caC | caT | 0031 | His | His |
| 2626c | Rv2626c_0126s | 0126 | gaC | gaT | 0042 | Asp | Asp |
| 2627c | Rv2627c_0063s | 0063 | gtC | gtT | 0021 | Val | Val |
| 2627c | Rv2627c_0230n | 0230 | cGa | cAa | 0077 | Arg | Gln |
| 2627c | Rv2627c_0310n | 0310 | Aga | Gga | 0104 | Arg | Gly |
| 2627c | Rv2627c_0431n | 0431 | aCg | aTg | 0144 | Thr | Met |
| 2627c | Rv2627c_0640n | 0640 | Ctt | Ttt | 0214 | Leu | Phe |
| 2627c | Rv2627c_0710n | 0710 | gAc | gGc | 0237 | Asp | Gly |
| 2627c | Rv2627c_0855s | 0855 | gaA | gaG | 0285 | Glu | Glu |
| 2627c | Rv2627c_1168n | 1168 | Cgg | Tgg | 0390 | Arg | Trp |
| 2628 | Rv2628_0004n | 0004 | Tcc | Ccc | 0002 | Ser | Pro |
| 2628 | Rv2628_0047n | 0047 | cCc | cTc | 0016 | Pro | Leu |
| 2628 | Rv2628_0063s | 0063 | ggC | ggT | 0021 | Gly | Gly |
| 2628 | Rv2628_0094n | 0094 | Cac | Tac | 0032 | His | Tyr |
| 2628 | Rv2628_0176n | 0176 | tCg | tTg | 0059 | Ser | Leu |
| 2628 | Rv2628_0203n | 0203 | cCt | cGt | 0068 | Pro | Arg |
| 2628 | Rv2628_0248n | 0248 | tTg | tGg | 0083 | Leu | Trp |
| 2628 | Rv2628_0286n | 0286 | Gcg | Acg | 0096 | Ala | Thr |
| 2686c | Rv2686c_0123s | 0123 | acC | acT | 0041 | Thr | Thr |
| 2686c | Rv2686c_0204s | 0204 | ccA | ccC | 0068 | Pro | Pro |
| 2686c | Rv2686c_0439s | 0439 | Ctg | Ttg | 0147 | Leu | Leu |
| 2687c | Rv2687c_0052n | 0052 | Cag | Aag | 0018 | Gln | Lys |
| 2687c | Rv2687c_0246s | 0246 | acG | acA | 0082 | Thr | Thr |
| 2687c | Rv2687c_0322n | 0322 | Gtg | Ttg | 0108 | Val | Leu |
| 2687c | Rv2687c_0559n | 0559 | Ctg | Gtg | 0187 | Leu | Val |
| 2687c | Rv2687c_0658n | 0658 | Cgg | Tgg | 0220 | Arg | Trp |
| 2687c | Rv2687c_560n | 0560 | cTg | cCg | 0187 | Leu | Pro |
| 2744c | Rv2744c_0045s | 0045 | ctG | ctA | 0015 | Leu | Leu |
| 2744c | Rv2744c_0186s | 0186 | ctC | ctT | 0062 | Leu | Leu |
| 2744c | Rv2744c_0457n | 0457 | Aag | Cag | 0153 | Lys | Gln |
| 2744c | Rv2744c_0487n | 0487 | Atg | Ctg | 0163 | Met | Leu |
| 2744c | Rv2744c_0688n | 0688 | Cgg | Tgg | 0230 | Arg | Trp |
| 2744c | Rv2744c_0689n | 0689 | cGg | cAg | 0230 | Arg | Gln |
| 2744c | Rv2744c_0755n | 0755 | cCc | cTc | 0252 | Pro | Leu |
| 2744c | Rv2744c_0808n | 0808 | Cag | Tag | 0270 | Gln | TER |
| 2873 | Rv2873_0203n | 0203 | gCg | gTg | 0068 | Ala | Val |
| 2873 | Rv2873_0429s | 0429 | ctC | ctT | 0143 | Leu | Leu |
| 2875 | Rv2875_0061n | 0061 | Gcg | Acg | 0021 | Ala | Thr |
| 2875 | Rv2875_0484n | 0484 | Agc | Cgc | 0162 | Ser | Arg |
| 2875 | Rv2875_0510s | 0510 | gtC | gtT | 0170 | Val | Val |
| 2875 | Rv2875_0543s | 0543 | acG | acA | 0181 | Thr | Thr |
| 2924c | Rv2924c_0098n | 0098 | gCt | gTt | 0033 | Ala | Val |
| 2924c | Rv2924c_0124n | 0124 | Gcg | Ccg | 0042 | Ala | Pro |
| 2924c | Rv2924c_0373s | 0373 | Ctg | Ttg | 0125 | Leu | Leu |
| 2924c | Rv2924c_0655n | 0655 | Gac | Cac | 0219 | Asp | His |
| 2924c | Rv2924c_0692n | 0692 | tCg | tGg | 0231 | Ser | Trp |
| 2924c | Rv2924c_0766n | 0766 | Gaa | Caa | 0766 | Glu | Gln |
| 2924c | Rv2924c_0844n | 0844 | Cga | Tga | 0844 | Arg | TER |
| 2949c | Rv2949c_0092n | 0092 | gTt | gGt | 0031 | Val | Gly |
| 2949c | Rv2949c_0363s | 0363 | caC | caT | 0121 | His | His |
| 2949c | Rv2949c_0375s | 0375 | ttC | ttT | 0125 | Phe | Phe |
| 2949c | Rv2949c_0442n | 0442 | Tcc | Acc | 0148 | Ser | Thr |
| 2949c | Rv2949c_0467n | 0467 | cGt | cAt | 0156 | Arg | His |
| 2952 | Rv2952_0028n | 0028 | Ctc | Ttc | 0010 | Leu | Phe |
| 2952 | Rv2952_0313n | 0313 | Gcc | Tcc | 0105 | Ala | Ser |
| 2952 | Rv2952_0454n | 0454 | Gtc | Atc | 0152 | Val | Ile |
| 2952 | Rv2952_0526n | 0526 | Ggg | Agg | 0176 | Gly | Arg |
| 2957 | Rv2957_0007n | 0007 | Cag | Tag | 0003 | Gln | TER |
| 2957 | Rv2957_0023n | 0023 | gCc | gTc | 0008 | Ala | Val |
| 2957 | Rv2957_0411s | 0411 | ctG | ctA | 0137 | Leu | Leu |
| 2957 | Rv2957_0447n | 0447 | ttC | ttA | 0149 | Phe | Leu |
| 2957 | Rv2957_0499n | 0499 | Tgc | Cgc | 0167 | Cys | Arg |
| 2957 | Rv2957_0705s | 0705 | aaG | aaA | 0235 | Lys | Lys |
| 2957 | Rv2957_0731n | 0731 | gGc | gAc | 0244 | Gly | Asp |
| 2958c | Rv2958c_0445n | 0445 | Atc | Gtc | 0149 | Ile | Val |
| 2958c | Rv2958c_0559n | 0559 | Ccg | Tcg | 0187 | Pro | Ser |
| 2958c | Rv2958c_0580n | 0580 | Tgt | Cgt | 0194 | Cys | Arg |
| 2958c | Rv2958c_0882s | 0882 | gtC | gtG | 0294 | Val | Val |
| 2959c | Rv2959c_0111s | 0111 | gtG | gtA | 0037 | Val | Val |
| 2959c | Rv2959c_0138s | 0138 | taC | taT | 0046 | Tyr | Tyr |
| 2959c | Rv2959c_0157n | 0157 | Aag | Gag | 0053 | Lys | Glu |
| 2959c | Rv2959c_0182n | 0182 | tGc | tTc | 0061 | Cys | Phe |
| 2959c | Rv2959c_0207n | 0207 | tgG | tgA | 0069 | Trp | TER |
| 2959c | Rv2959c_0219n | 0219 | gaG | gaT | 0073 | Glu | Asp |
| 2959c | Rv2959c_0438n | 0438 | atT | atG | 0146 | Ile | Met |
| 2959c | Rv2959c_0618s | 0618 | ggA | ggC | 0206 | Gly | Gly |
| 2959c | Rv2959c_0670n | 0670 | Cat | Gat | 0224 | His | Asp |
| 2962c | Rv2962c_0066s | 0066 | gtG | gtA | 0022 | Val | Val |
| 2962c | Rv2962c_0494n | 0494 | tGg | tAg | 0165 | Trp | TER |
| 2962c | Rv2962c_0711s | 0711 | gcT | gcC | 0237 | Ala | Ala |
| 2962c | Rv2962c_0817s | 0817 | Ctg | Ttg | 0273 | Leu | Leu |
| 2962c | Rv2962c_0880n | 0880 | Caa | Taa | 0294 | Gln | Ter |
| 2962c | Rv2962c_0922n | 0922 | Atc | Gtc | 0308 | Ile | Val |
| 2962c | Rv2962c_1168n | 1168 | Cgg | Tgg | 0390 | Arg | Trp |
| 2962c | Rv2962c_1294n | 1294 | Gag | Tag | 0432 | Glu | TER |
| 2962c | Rv2962c_1300n | 1300 | Gct | Cct | 0434 | Ala | Pro |
| 2985 | Rv2985_0018s | 0018 | tcG | tcA | 0006 | Ser | Ser |
| 2985 | Rv2985_0792n | 0792 | aaG | aaC | 0264 | Lys | Asn |
| 3129 | Rv3129_0095n | 0095 | gAc | gCc | 0032 | Asp | Ala |
| 3132c | Rv3132c_0019n | 0019 | Gtc | Atc | 0007 | Val | Ile |
| 3132c | Rv3132c_0147s | 0147 | gaT | gaC | 0049 | Asp | Asp |
| 3132c | Rv3132c_0626n | 0626 | gCt | gTt | 0209 | Ala | Val |
| 3132c | Rv3132c_0660s | 0660 | cgT | cgC | 0220 | Arg | Arg |
| 3132c | Rv3132c_0848n | 0848 | aTt | aCt | 0283 | Ile | Thr |
| 3132c | Rv3132c_1068s | 1068 | gaG | gaA | 0356 | Glu | Glu |
| 3132c | Rv3132c_1440s | 1440 | ggT | ggC | 0480 | Gly | Gly |
| 3132c | Rv3132c_1680s | 1680 | gcG | gcC | 0560 | Ala | Ala |
| 3133c | Rv3133c_0419n | 0419 | gCc | gGc | 0140 | Ala | Gly |
| 3133c | Rv3133c_0517s | 0517 | Cga | Aga | 0174 | Arg | Arg |
| 3221c | Rv3221c_0030s | 0030 | gtG | gtA | 0010 | Val | Val |
| 3221c | Rv3221c_0085n | 0085 | Gtc | Atc | 0029 | Val | Ile |
| 3221c | Rv3221c_0186n | 0186 | caG | caT | 0062 | Gln | His |
| 3261 | Rv3261_0015s | 0015 | gtT | gtC | 0005 | Val | Val |
| 3261 | Rv3261_0390s | 0390 | gaG | gaA | 0130 | Glu | Glu |
| 3261 | Rv3261_0416n | 0416 | gGc | gAc | 0139 | Gly | Asp |
| 3261 | Rv3261_0905n | 0905 | aCg | aTg | 0302 | Thr | Met |
| 3262 | Rv3262_0639s | 0639 | ccG | ccA | 0213 | Pro | Pro |
| 3262 | Rv3262_1031n | 1031 | gCa | gTa | 0344 | Ala | Val |
| 3262 | Rv3262_1095s | 1095 | cgC | cgG | 0365 | Arg | Arg |
| 3354 | Rv3354_0162s | 0162 | gtC | gtG | 0054 | Val | Val |
| 3354 | Rv3354_0335n | 0335 | gTc | gCc | 0112 | Val | Ala |
| 3407 | Rv3407_0250n | 0250 | Cgc | Tgc | 0084 | Arg | Cys |
| 3547 | Rv3547_0144s | 0144 | ctG | ctT | 0048 | Leu | Leu |
| 3547 | Rv3547_0268n | 0268 | Ctc | Gtc | 0090 | Leu | Val |
| 3547 | Rv3547_0337n | 0337 | Gac | Aac | 0113 | Asp | Asn |
| 3589 | Rv3589_0051s | 0051 | tcA | tcC | 0017 | Ser | Ser |
| 3589 | Rv3589_0557n | 0557 | gAg | gCg | 0186 | Glu | Ala |
| 3589 | Rv3589_0710n | 0710 | gCc | gAc | 0237 | Ala | Asp |
| 3763 | Rv3763_0148n | 0148 | Ccg | Acg | 0050 | Pro | Thr |
| 3763 | Rv3763_0468s | 0468 | gtG | gtA | 0156 | Val | Val |
| 3763 | Rv3763_0472n | 0472 | Tgt | Agt | 0158 | Cys | Ser |
| 3804c | Rv3804c_0012s | 0012 | gtT | gtC | 0004 | Val | Val |
| 3804c | Rv3804c_0315s | 0315 | gaC | gaT | 0105 | Asp | Asp |
| 3804c | Rv3804c_0433n | 0433 | Acc | Gcc | 0145 | Thr | Ala |
| 3804c | Rv3804c_0464n | 0464 | aAc | aGc | 0155 | Asn | Ser |
| 3804c | Rv3804c_0933s | 0933 | ggC | ggG | 0311 | Gly | Gly |
| 3872 | Rv3872_0020n | 0020 | gAt | gGt | 0007 | Asp | Gly |
| 3872 | Rv3872_0295n | 0295 | Gaa | Taa | 0099 | Glu | TER |
| 3874 | Rv3874_0126s | 0126 | caG | caA | 0042 | Gln | Gln |
| 3874 | Rv3874_0202n | 0202 | Gaa | Aaa | 0068 | Glu | Lys |
| 3875 | Rv3875_0009n | 0009 | gaG | gaT | 0003 | Glu | Asp |
| 3875 | Rv3875_0015s | 0015 | caG | caA | 0005 | Gln | Gln |
| 3875 | Rv3875_0218n | 0218 | gCg | gTg | 0073 | Ala | Val |
| 3875 | Rv3875_0220n | 0220 | Cgg | Tgg | 0074 | Arg | Trp |
| 3890c | Rv3890c_0166n | 0166 | Cag | Aag | 0056 | Gln | Lys |
| 3890c | Rv3890c_0259n | 0259 | Acc | Gcc | 0087 | Thr | Ala |
| 3891c | Rv3891c_0145n | 0145 | Acc | Gcc | 0049 | Thr | Ala |
| 3891c | Rv3891c_0234s | 0234 | ggC | ggT | 0078 | Gly | Gly |
| 3904c | Rv3904c_0239n | 0239 | gGc | gTc | 0080 | Gly | Val |
| 3904c | Rv3904c_0244n | 0244 | Atg | Gtg | 0082 | Met | Val |
| 3905c | Rv3905c_0079n | 0079 | Cat | Tat | 0027 | His | Tyr |
| 3905c | Rv3905c_0153s | 0153 | ggC | ggT | 0051 | Gly | Gly |
| 3905c | Rv3905c_0173n | 0173 | tGg | tAg | 0058 | Trp | TER |
| 3905c | Rv3905c_0279s | 0279 | tcG | tcC | 0093 | Ser | Ser |
| 3908 | Rv3908_0022n | 0022 | Aaa | Gaa | 0008 | Lys | Glu |
| 3908 | Rv3908_0142n | 0142 | Cgg | Ggg | 0048 | Arg | Gly |
| 3908 | Rv3908_0390s | 0390 | ctC | ctT | 0130 | Leu | Leu |
| 3908 | Rv3908_0556n | 0556 | Gcc | Ccc | 0186 | Ala | Pro |
| 3908 | Rv3908_0615s | 0615 | caG | caA | 0205 | Gln | Gln |
