# Supplementary material for: High Functional Diversity in Mycobacterium tuberculosis Driven by Genetic Drift and Human Demography
Source: PLoS Biol. 2008 Dec 16;6(12):e311. doi: 10.1371/journal.pbio.0060311 (PMC2602723; doi:10.1371/journal.pbio.0060311)
Supplement: Supplementary file 9 [file pbio.0060311.st006.doc]

**Table S6.** Non-synonymous and synonymous single nucleotide polymorphisms identified in 107 *M. tuberculosis* complex strains by gene category, phylogenetic clade, and branch in the phylogenetic tree (Figure 1).

|  | **nSNPs** | **sSNPs** | **nSNPs : sSNPs** | **95% confidence interval** |
| --- | --- | --- | --- | --- |
| *Total* | 231 | 139 | 1.66 | (1.34 - 2.07) |
| *Genes* |  |  |  |  |
| Housekeeping | 94 | 59 | 1.59 | (1.14 - 2.25) |
| Virulence | 46 | 23 | 2.00 | (1.19 - 3.46) |
| Surface | 91 | 57 | 1.60 | (1.13 - 2.27) |
| *Clades* |  |  |  |  |
| ‘Ancient’ Human | 77 | 50 | 1.54 | (1.07 - 2.25) |
| ‘Modern’ Human | 107 | 57 | 1.88 | (1.35 - 2.64) |
| Animal | 40 | 28 | 1.43 | (0.86 - 2.40) |
| *Branches* |  |  |  |  |
| Internal | 100 | 66 | 1.52 | (1.10 - 2.10) |
| External | 131 | 73 | 1.79 | (1.34 - 2.42) |
